# A tissue atlas of ulcerative colitis revealing evidence of sex-dependent differences in disease-driving inflammatory cell types and resistance to TNF inhibitor therapy

**DOI:** 10.1126/sciadv.add1166

**Published:** 2023-01-20

**Authors:** Aaron T. Mayer, Derek R. Holman, Anav Sood, Utkarsh Tandon, Salil S. Bhate, Sunil Bodapati, Graham L. Barlow, Jeff Chang, Sarah Black, Erica C. Crenshaw, Alexander N. Koron, Sarah E. Streett, Sanjiv S. Gambhir, William J. Sandborn, Brigid S. Boland, Trevor Hastie, Robert Tibshirani, John T. Chang, Garry P. Nolan, Christian M. Schürch, Stephan Rogalla

**Affiliations:** ^1^ Department of Bioengineering, Stanford University School of Medicine, Stanford, CA, USA.; ^2^ Molecular Imaging Program at Stanford, Department of Radiology, Stanford University School of Medicine, Stanford, CA, USA.; ^3^ Enable Medicine LLC, Menlo Park, CA, USA.; ^4^ Division of Gastroenterology and Hepatology, Department of Medicine, Stanford University School of Medicine, Stanford, CA, USA.; ^5^ Department of Biomedical Data Science and of Statistics, Stanford University School of Medicine, Stanford, CA, USA.; ^6^ Department of Microbiology and Immunology, Stanford University School of Medicine, Stanford, CA, USA.; ^7^ Division of Gastroenterology, Department of Medicine, University of California San Diego, La Jolla, CA, USA.; ^8^ Department of Pathology, Stanford University School of Medicine, Stanford, CA, USA.; ^9^ Department of Pathology and Neuropathology, University Hospital and Comprehensive Cancer Center Tübingen, Tübingen, Germany.

## Abstract

Although literature suggests that resistance to TNF inhibitor (TNFi) therapy in patients with ulcerative colitis (UC) is partially linked to immune cell populations in the inflamed region, there is still substantial uncertainty underlying the relevant spatial context. Here, we used the highly multiplexed immunofluorescence imaging technology CODEX to create a publicly browsable tissue atlas of inflammation in 42 tissue regions from 29 patients with UC and 5 healthy individuals. We analyzed 52 biomarkers on 1,710,973 spatially resolved single cells to determine cell types, cell-cell contacts, and cellular neighborhoods. We observed that cellular functional states are associated with cellular neighborhoods. We further observed that a subset of inflammatory cell types and cellular neighborhoods are present in patients with UC with TNFi treatment, potentially indicating resistant niches. Last, we explored applying convolutional neural networks (CNNs) to our dataset with respect to patient clinical variables. We note concerns and offer guidelines for reporting CNN-based predictions in similar datasets.

## Teaser

Ulcerative colitis tissue atlas reveals evidence of sex-dependent differences in inflammation and TNF inhibitor resistance.

## INTRODUCTION

Ulcerative colitis (UC), a chronic relapsing inflammatory bowel disease (IBD), is one of the most common autoimmune disorders (*1*, *2*). UC results from a dysregulated interplay of genetic, immune, environmental, and microbiome factors (*1*–*3*). It can persist for decades, if not afflicting patients for a lifetime, resulting in high medical costs, diminished workplace productivity, and substantially impaired quality of life. Because of the chronic inflammation associated with UC, patients have a 1.5 times higher long-term risk for colorectal cancer compared to healthy individuals (*4*, *5*). In recent years, novel treatment options for UC have been introduced, expanding the therapeutic standard of care from broadly immunosuppressive monotherapies (i.e., corticosteroids and azathioprine) to include more targeted immunomodulatory drugs. The expansion of available treatment options requires a shift toward a precision medicine approach. This shift is necessary to address the sensitivity of treatment selection to underlying patient UC–associated variables. For example, sex-dependent differences in UC presentation are increasingly noted, manifesting in sex-dependent prevalence rates, choice of therapy, and chemically induced disease progression in animal models (*6*–*8*).

Tumor necrosis factor inhibitors (TNFis) such as infliximab and adalimumab are frontline targeted therapies for UC and lead to increased rates of sustained remission and decreased rates of surgery (*9*, *10*). These inhibitors have fewer side effects than standard immunosuppressants, and the incidence of chronic inflammation-induced colorectal cancer is reduced in TNFi-treated patients; however, TNFi-treated patients have an increased risk for opportunistic infections and for some malignancies including lymphomas, acute myeloid leukemia, myelodysplastic syndromes, and skin carcinomas (*11*). Furthermore, primary nonresponse to TNFi therapy occurs in 13 to 40% of patients with UC, and loss of response or adverse effects are reported in up to 46% of the remaining patients within 12 months of therapy initiation (*12*–*15*). Moreover, the introduction of TNFi to standard UC treatment regimens results in substantially increased costs. Combined direct and indirect costs caused by the disease are estimated at $45 billion per year in the United States and Europe (*1*). Additional biologics, such as vedolizumab, an antibody that antagonizes α4β7 integrins, have been approved for the treatment of patients with UC, but identification of patients who will respond to TNFi remains an unmet clinical challenge (*9*, *16*). Predictive biomarkers to identify the best therapy for each individual patient at the time of diagnosis are lacking (*15*–*17*). All of the above suggests that UC is a complex, heterogeneous disease and underlines the need for biomarkers to guide UC therapy.

The success of clinical and biological phenotyping in identifying novel biomarkers to guide UC therapy has been limited, although single-cell omics studies have identified some promising biomarkers in the closely related Crohn’s disease. The fecal proteins calprotectin and lactoferrin, which are released by activated neutrophils in the gut, are useful in monitoring disease activity but have no role in the decision-making process with regard to a specific treatment (*10*, *18*). Serological markers such as C-reactive protein and anti-neutrophil cytoplasmic antibodies were investigated as therapy predictors in Crohn’s disease, but data were inconclusive. Studies suggest that the only reliable clinical predictor of TNFi therapy failure in IBD was the presence of strictures in Crohn’s disease as assessed endoscopically or radiologically; however, this may be an indicator of disease severity and complications rather than underlying disease biology (*19*). Therefore, recent studies have aimed at enabling precision medicine in IBD through the use of new technologies like single-cell RNA sequencing (scRNA-seq) and mass cytometry to predict therapy response and disease activity (*20*–*22*). scRNA-seq analysis in a cohort of Crohn’s disease patients with ileal disease was able to identify pathogenic cellular modules that were weakly, although significantly, associated with resistance to TNFi therapy (*21*). The presence of these immune cell modules suggests a spatial and functional relationship between the underlying cell types, but spatial information is lost during the preparation of samples for scRNA-seq, so this has not been tested.

Imaging technologies capable of spatially mapping immune cell states within the tissue at single-cell resolution should improve our understanding of how cellular interactions and gut remodeling contribute to UC pathogenesis and TNFi resistance. Multiplexed imaging tools are revolutionizing the development of biomarker-based diagnostics and have led to novel therapeutic insights in other disease settings (*23*). Imaging mass cytometry has been used to better understand the high-dimensional tissue pathology in patients with breast cancer and the changing cell populations associated with diabetes disease progression and, more recently, also to dissect the immune and epithelial cell interactions in IBD (*24*–*26*). A multiplexed immunofluorescence-based imaging technology called CO-Detection by indEXing (CODEX) has allowed simultaneous visualization of up to 60 biomarkers on intact tissue with single-cell resolution (*27*, *28*). These biomarker panels can be custom designed and used to elucidate cell types and their functional states. The spatial interplay between these markers can be used to identify cell-cell interactions and cellular neighborhoods (CNs). In colorectal cancer, these spatial features have revealed coordinated CNs underlying antitumoral immunity and disease progression. These changes in the tumor-immune microenvironment were correlated with patient outcomes but were not visible as changes at the single-cell level (*28*). Similarly, these representations of spatial features were used in a second study to identify biomarkers of pembrolizumab therapy response in patients with cutaneous T cell lymphoma (*29*).

Here, we used CODEX to create a spatial atlas of the gut and its rewiring during UC pathogenesis. In 42 biopsy regions taken from a cohort of 34 individuals, we analyzed 52 biomarkers on 1,710,973 spatially resolved single cells to identify 13 conserved cell types. We mapped changes in frequency and functional states during inflammation and in patients with TNFi treatment. By using Voronoi representations of our data, we were able to represent it at multiple spatial scale cell frequencies, cell-cell pairwise interactions, and CNs. These representations allowed us to identify cellular niches that may respond to or remain unchanged in the face of TNFi therapy. We observe sex differences in the relative abundance of these niches in our patient cohort. However, our data suggest that these TNFi-responsive or TNFi-resistant niches have only minimal predictive value when determining whether the patient as a whole is likely to respond to subsequent TNFi therapy. To increase ease of user accessibility to our results, we created a cloud-based platform called the Explorer that allows users to search our spatial maps for additional insights into UC. Our research suggests that examining cells within their spatial context may offer insights into developing biomarkers for therapy response in UC and that this framework has the potential to improve our understanding of inflammatory immune diseases.

## RESULTS

### CODEX creates a spatial atlas of the UC inflammatory microenvironment

**Fig. 1.**
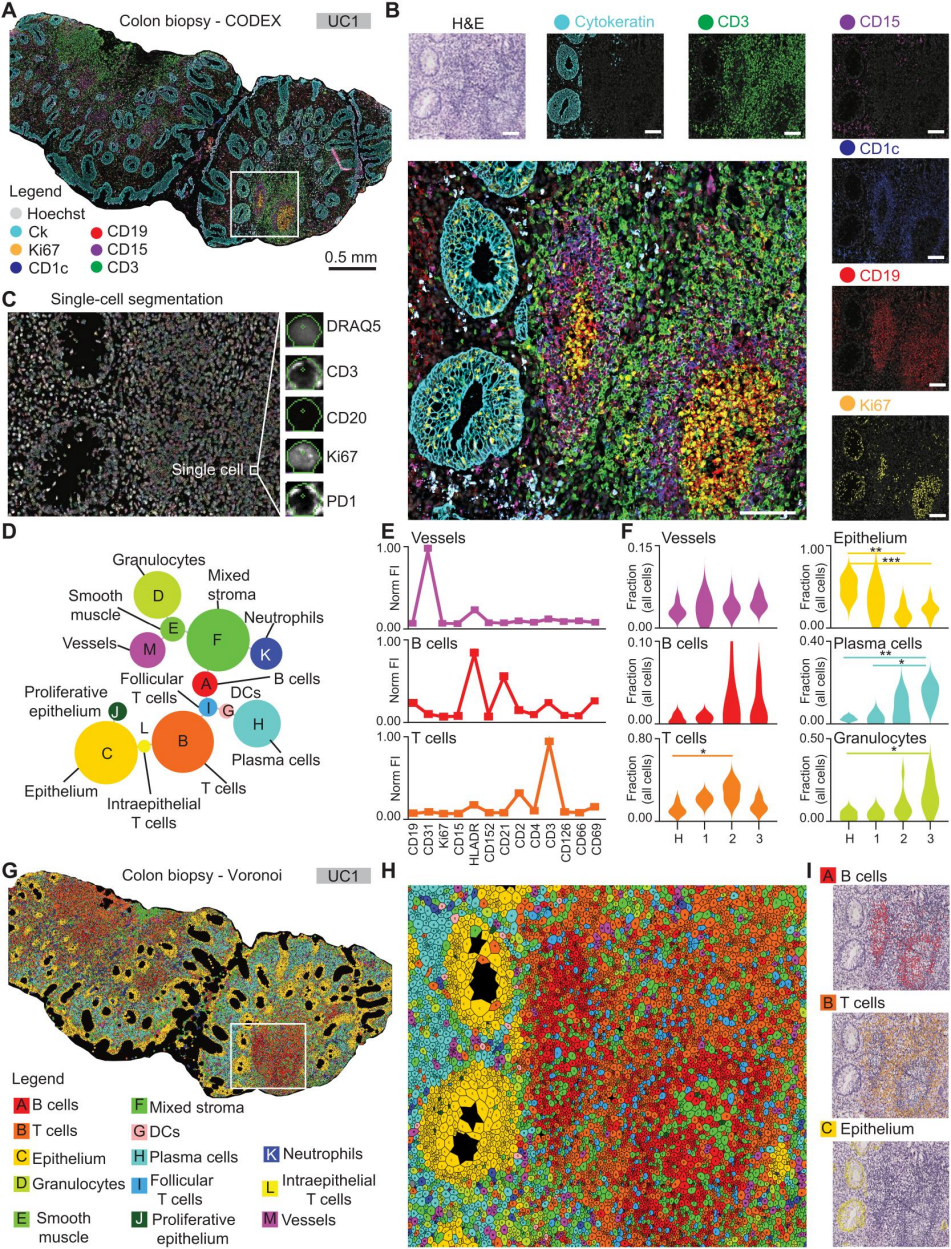
A spatial atlas of the UC inflammatory microenvironment. (**A**) CODEX image with Hoechst nuclear stain (gray), cytokeratin (cyan), Ki67 (yellow), CD1c (blue), CD19 (red), CD15 (purple), and CD3 (green) shown as a seven-color composite image selected from five markers stained on the same tissue section. (**B**) Zoomed-in view of the region inside the white box in (A) shown as a seven-color composite image (large panel) and as hematoxylin and eosin (H&E) and two-color images of Hoechst and each indicated marker individually (small panels). (**C**) Representative example of single-cell segmentation within the same box with DRAQ5 nuclear staining (gray) and the segmentation masks overlaid (colored). (**D**) Minimum spanning tree of the cell-type clusters identified in this study. Nodes represent clusters with sizes indicative of the number of cells across all samples, and distances between nodes indicate their relationships in high-dimensional marker space, not physical space. (**E**) Examples of marker expression profiles, in normalized fluorescent intensity (Norm FI) were extracted and used for the identification of cell types. (**F**) Single-cell clustering results and cell types across the entire cohort, evaluated at the patient level. H, healthy controls; 1 to 3, patients with UC with Mayo scores 1 to 3, respectively. Statistics: Student’s *t* test with Bonferroni correction for multiple comparisons (diseased versus healthy); *P < 0.05, **P < 0.01, and ***P < 0.001. (**G** and **H**) Voronoi representations of the identified cell types on the same tissue section as shown in (A) and (B). (G) Biopsy overview and legend of cell types. (H) Zoomed-in view of the region denoted in the white box in (G). (**I**) Example mapping of clustering results to H&E images used to validate cell type identity “A” (B cells, top), “B” (T cells, middle), and “C” (epithelium, bottom).

We used CODEX to characterize the inflammatory microenvironment of the colon in 42 tissue biopsy regions from 29 patients with UC and five healthy controls. Fifteen patients with UC were being treated with TNFi at the time of biopsy (fig. S1A and table S1). Long-term clinical follow-up was available for all patients, allowing categorization of the patients into TNFi responders versus nonresponders following the practice guidelines of the American Gastroenterological Association for moderate to severe UC (*10*). In line with previous reports (*12*, *30*), in our cohort, ~50% of TNFi-treated patients did not subsequently respond to TNFi therapy (table S1). We imaged multiple tissue regions per sample with a 52-plex CODEX antibody panel including markers for immune, epithelial, and stromal cell components of the intestine as well as functional markers such as 4-1BB [also known as TNF receptor superfamily member 9 (TNFRSF9) or CD137], interleukin-6R (also known as CD126), and TNFR2 (also known as CD120b) (Fig. 1, A and B; fig. S1, B to J; and table S2). After CODEX imaging, we performed hematoxylin and eosin (H&E) staining on the same tissue sections. We observed excellent morphological correlation between fluorescent markers and H&E images, exemplified by cytokeratin positivity in the epithelium, CD19 staining of the B cell follicles and CD1c expression in the associated antigen-presenting cells, CD3 staining in the surrounding T cell zone, CD15-positive clusters of granulocytes, and Ki67 staining of the proliferating cells in germinal centers and basal epithelia (Fig. 1B). Cellular segmentation and fluorescent marker quantification resulted in spatially resolved single-cell data that were used for downstream analysis (Fig. 1C). Using unsupervised X-shift clustering (*23*) and manual cluster curation based on marker expression profiles, tissue localization, and tissue morphology, we identified 13 distinct cell-type clusters in our samples (Fig. 1, D and E; figs. S2 and S3; and Materials and Methods). These included eight distinct immune cell clusters and two epithelial clusters as well as smooth muscle, mixed stroma, and vasculature clusters. The immune cell clusters consisted of adaptive and innate immune cell types, including three types of T cells, B cells, plasma cells, and dendritic cells (DCs), neutrophils, and granulocytes (Fig. 1, D and E, and figs. S2 and S3). We compared unsupervised clustering results to manual gating of similar cell populations based on the marker expression profiles, and the frequencies of cell types identified by both methods showed a strong correlation (fig. S4, A to C). Of these cell types, we observed Mayo disease severity score–dependent, statistically significant changes in the frequencies of T cells, epithelium, plasma cells, granulocytes, DCs, and intraepithelial T cells (Fig. 1F and fig. S4C). For downstream analyses, we chose cell types identified by unsupervised clustering. To verify the clusters and assess their spatial distribution in the tissue, we graphically represented the clustering results as Voronoi diagrams colored by cell type and overlaid selected clusters on the H&E-stained images (Fig. 1, G to I). In summary, by CODEX tissue imaging and computational single-cell identification, we created a high-dimensional spatial atlas of the inflammatory microenvironment in patients with UC treated or not with TNFi, which can be used for biomarker discovery and clinical correlation using a cloud-based online tool that we call Explorer (https://app.enablemedicine.com/uc-study).

### Immune cell contacts reorganize gut tissue architecture and contribute to UC disease heterogeneity

**Fig. 2.**
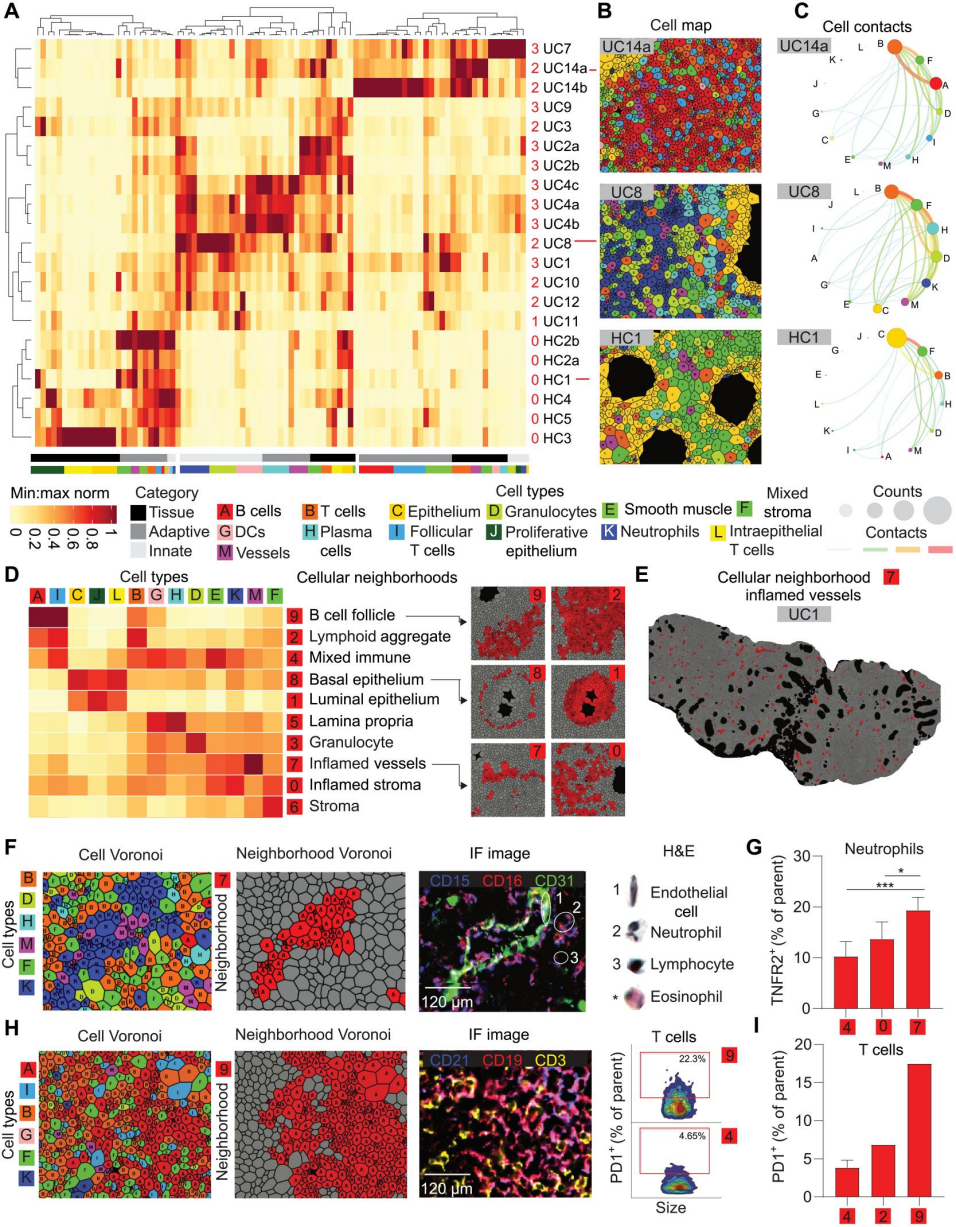
UC immune cell contacts disrupt gut architecture and generate inflammatory niches. (**A**) Heatmap depicting cell-cell contacts in healthy controls (HCs; *n* = 6 tissues, five individuals) and TNFi-naïve patients with UC (UC; *n* = 15 tissues, 11 patients). Columns represent normalized pairwise cell-cell contacts, and rows represent samples. The number of clusters was selected to highlight healthy controls versus patients with UC. Grayscale bars represent the frequencies of cell-cell contacts categorized as tissue contacts (e.g., epithelial/stromal/vessel), adaptive immune contacts (e.g., T cells and B cells), and innate immune contacts (e.g., granulocytes). The bars colored by cell type represent the relative frequencies of each contact pair for every cell type. (**B** and **C**) Representative examples of (B) cell-type Voronoi diagrams and (C) circular connectivity diagrams depicting the frequencies of cell types and pairwise cell-cell contacts. (**D**) Heatmap of 10 distinct cellular neighborhoods (CNs) based on spatial enrichment of the 13 cell-type clusters. Intensity scale identical to (A). (**E** and **F**) CN-7 (inflamed vessels) (E) mapped on patient biopsy UC1 and (F) depicted alongside cell-type Voronoi diagram and three-color immunofluorescent image with CD15 (blue), CD16 (red), and CD31 (green). Selected cell types are shown in corresponding H&E images on the right. The asterisk indicates an eosinophil taken from a different tissue region for comparison. (**G**) The frequency of neutrophils expressing TNFR2 in CN-0 (inflamed stroma) versus CN-7 (inflamed vessels) and CN-4 (mixed immune). Statistics: Student’s *t* test, *P < 0.05 and ***P < 0.01 (*n* = 5). (**H**) Example of CN-9 (B cell follicle) depicted alongside its corresponding cell-type Voronoi diagram and composite immunofluorescent image with CD21 (blue), CD19 (red), and CD3 (yellow). Biaxial plots depict PD1^+^ T cells as a proportion of all T cells in CN-9 versus CN-4 (mixed immune). (**I**) Bar plot depicting trend for increased PD1^+^ T cells in CN-9 versus CN-2 (lymphoid aggregate) and CN-4 in untreated patients with UC (*n* = 5).

The frequencies of epithelial, stromal, and immune cell types in the colonic microenvironment, as well as their spatial organization, should provide insights into how various cell types and their interactions influence UC development, progression, and therapeutic response. A simple way of analyzing the spatial organization of cell types in tissue is to compute their pairwise contacts. Analysis of pairwise contacts in images of samples from healthy controls and 14 patients who had not been treated with a TNFi at the time of biopsy revealed that a spectrum of colonic and immune cell contacts contribute to tissue organization (Fig. 2A). The healthy controls clustered together and were defined by enriched contacts between epithelial cells and stromal cells (Fig. 2A, six bottom rows). Cellular contacts in patient with UC samples showed substantial variability that was not strongly associated with Mayo score, resulting in two clusters (rows 1 to 3 and 4 to 15). In most patient samples, we observed relative enrichment for paired cell-cell contacts between innate immune cells (e.g., patient UC8; Fig. 2A, center rows), whereas, in a minority of patient samples, we observed enrichment for contacts between adaptive immune cells (e.g., patient UC14a; Fig. 2A, top three rows). We visualized the cell-type clusters for each patient using Voronoi diagrams (Fig. 2B) and plotted the frequencies of pairwise cell-cell contacts using circular “connectivity” diagrams (Fig. 2C) (*27*). The most frequent cell-type clusters and contacts in patients with UC were immune related. For example, in patient UC8, T cells were the most frequent cell type, followed by stroma, plasma cells, granulocytes, neutrophils, and vasculature. Epithelial cells were only the seventh most abundant cell-type cluster, which reflects their destruction and replacement by the immune infiltrate during active UC (Fig. 2, B and C, middle panels). The cell-cell connectivity map for patient UC14a revealed T cells predominantly connected to B cells and stroma, whereas cell-cell contacts to and in-between innate immune cell types, especially neutrophils, were less frequent (Fig. 2, B and C, top panels). We hypothesized that the observed heterogeneity between cell-cell contacts in patients with UC contributes to differences in higher-order tissue organization and diverging disease states and therapy responses.

### Characteristic CNs of the UC immune microenvironment are conserved across patients and are associated with immune cell functional state

We previously showed that, in addition to pairwise cell-cell contacts, higher-order tissue structures such as CNs provide important information with regard to disease status and therapy response (*28*). CNs can be seen as functional subunits inside intact tissue. We performed CN analysis on the entire dataset of patient with UC and identified 10 CNs common to all patients (Fig. 2D). Among these was a CN highly enriched in B cells and follicular T cells, which we termed the B cell follicle (CN-9); a CN moderately enriched in B cells, follicular T cells, and T cells, termed lymphoid aggregate (CN-2); a mixed immune CN (CN-4); a basal epithelium CN enriched in epithelial cells, proliferating (Ki67^+^) epithelial cells, intraepithelial T cells, and other immune cells (CN-8); a luminal epithelium CN with a composition similar to CN-8 but spatially distinct (CN-1); a lamina propria CN enriched in plasma cells and DCs (CN-5); a granulocyte-enriched CN (CN-3); a CN enriched in the vasculature, smooth muscle, and neutrophils, which we termed inflamed vasculature (CN-7); a CN enriched in the stroma, neutrophils, and other immune cells, which we termed inflamed stroma (CN-0); and a noninflamed stroma CN (CN-6) (Fig. 2, D and E). Significant increases were seen in granulocyte (CN-3), mixed immune (CN-4), and lamina propria (CN-5) neighborhoods in association with increasing Mayo score. Unexpectedly, the lymphoid aggregate (CN-2) neighborhood increased through Mayo score 2 and then decreased from Mayo score 2 to 3. Significant decreases were observed for luminal epithelium (CN-1) and basal epithelium (CN-8) as Mayo score increased, consistent with healthy gut tissue destruction and the trends observed at the cell frequency level (fig. S5).

In addition to determining the frequencies of CNs across patient groups, we also analyzed their “functional states” as previously described (*28*). CN functional states are determined on the basis of the local enrichment with one or more specific cell types expressing certain functional markers, and their analysis provides a measure of how a given CN’s characteristics influence cellular function during disease progression and treatment. Specifically, we were interested in how TNFi treatment influenced the distribution of TNFR2 on various cell types and how this affected functional states. We found that CN-7 (inflamed vasculature) had a significantly higher frequency of TNFR2^+^ neutrophils as compared to CN-0 and CN-4, the two other neighborhoods enriched the most for neutrophils (Fig. 2, F and G). Similarly, programmed cell death-1 (PD-1)^+^ T cell frequencies varied greatly depending on whether a T cell was found in a B cell follicle neighborhood (CN-9), a lymphoid aggregate (CN-2), or a mixed immune neighborhood (CN-4) (Fig. 2, H and I). This suggests an important role for CNs in the functional states of individual cells.

### Innate immune cell populations may persist during TNFi therapy

**Fig. 3.**
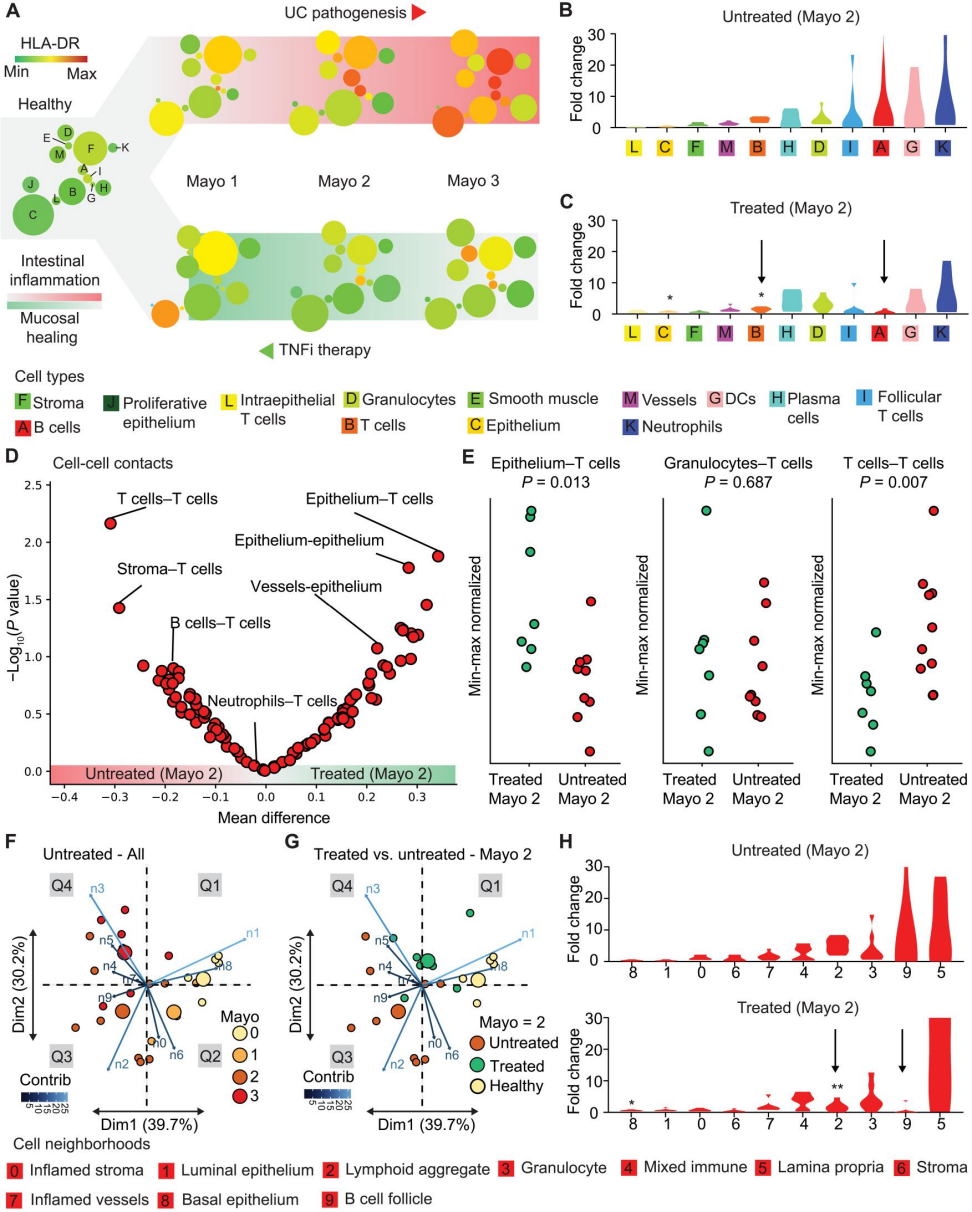
TNFi therapy shifts adaptive immune cells, contacts, and neighborhoods toward homeostasis. (**A**) A scaffold depicting minimum spanning tree representations of cell populations and biomarker expression, e.g. HLA-DR (human leucocuyte antigen DR isotype) along trajectories of UC pathogenesis and healing during TNF therapy. Node size represents the average frequency of each cell population for each cohort, and color represents the relative expression of TNFRSF9, a biomarker of immune activation. Color scale: green, low; red, high. Cluster D (granulocytes) appears to have high expression of the depicted marker in all subgroups, but this is due to the known propensity for antibodies to nonspecifically bind to this population of cells. This population was therefore excluded from further biomarker expression analysis. (**B** and **C**) Fold change of cell-type frequency relative to healthy controls for Mayo 2 patients (B) without or (C) with TNFi therapy. Statistics treated versus untreated: *t* test, *P < 0.05. Arrows indicate T cells (B) and B cells (A). (**D**) Volcano plot of cell-cell contact enrichment in Mayo 2 patients with versus without TNFi therapy. (**E**) T cell contact frequencies with epithelium, granulocytes, or other T cells in treated versus untreated Mayo 2 patient cohorts. Statistics: Student’s *t* test. (**F**) Principal components analysis based on frequency vector of cell neighborhoods for each untreated patient colored by Mayo score. Neighborhood weights (magnitude and direction) are overlaid onto principal component dimension 1 (Dim1) and dimension 2 (Dim2). Large circles represent the centroid for each Mayo score (or control). (**G**) Treated Mayo 2 patients projected onto the same principal components as computed in (F). (**H**) Fold changes of cell neighborhood frequencies relative to healthy controls for Mayo 2 patients without (top) or with (bottom) TNFi therapy. Statistics: *t* test, *P < 0.05 and **P < 0.01.

Identification of the biological basis for the resistance to TNFi might allow for a priori identification of patients with UC who will be resistant to TNFi treatment and inform the selection of alternative therapy. Although we lacked longitudinal samples, biopsies taken from the same patient before and after treatment, we were able to compare biopsies from treated and untreated patients with identical Mayo severity scores. To identify potential mechanisms that may underlie resistance to TNFi, we created a framework to assess architectural changes associated with gut inflammation during disease progression and mucosal healing in patients with TNFi treatment. These architectural changes were first depicted using minimum spanning trees, where average marker expression is visualized as a function of cell type. We observed that the expression level of the costimulatory TNFR 4-1BB (also known as TNFRSF9 or CD137), a marker of T cell activation implicated in UC inflammation (*31*), increased with disease severity, and levels decreased in treated patients except for in neutrophils and proliferative epithelium (Fig. 3A). Architectural changes were further quantified using cell-type frequency. TNFi responsiveness was primarily defined by mucosal healing (e.g., epithelium recovery) (Fig. 3A and fig. S6). Patients with TNFi therapy had substantial changes in the frequencies of adaptive immune cell types (Fig. 3, B and C). Specifically, we observed statistically significant reductions in T cell frequency and increases in the epithelium in treated versus untreated Mayo 2 patients. Although B cell levels appeared to decrease upon treatment, these differences did not reach statistical significance due to the high variability of B cell frequencies in patients with UC. Compared to their adaptive counterparts, there were no strong trends or significant changes in frequencies of innate immune cells in the face of TNF blockade.

### TNFi therapy is associated with altered adaptive immune cell contacts

Given the observation that patients with TNFi therapy had decreased adaptive immune cell frequencies such as T cells and B cells, we sought to determine whether the treated patients exhibited altered immune cell contacts. We observed altered contacts between T cells and other adaptive immune cells (e.g., T cell–T cell and T cell–B cell). This was similarly true for contacts between T cells with gut stroma and epithelium (Fig. 3, D and E). In contrast, T cell contacts with innate immune cells such as granulocytes were completely unchanged by treatment (Fig. 3, D and E). Homotypic innate immune cell contacts such as granulocyte-granulocyte contacts did not differ with or without TNFi therapy either. This rewiring of only certain immune cell contacts suggested that TNFi treatment has spatially dependent effects on inflammatory CNs in the gut.

### Innate immune neighborhoods are present in TNFi-treated patients

We next examined changes to CNs associated with disease severity, progression, and recovery to determine whether observed changes in cell frequencies and cell-cell contacts are correlated with higher-order architectural changes. To visualize the reorganization of CNs during disease progression and therapy, we performed principal components analysis on the CN frequencies for healthy controls and untreated patients to map the trajectories associated with disease progression (Fig. 3F). When CN weights were plotted onto principal components 1 and 2, cluster centroids separated into roughly four quadrants, with disease severity increasing on average along a clockwise trajectory. Healthy controls clustered in the top right quadrant and were associated with basal and luminal epithelium (CN-8 and CN-1, respectively). Mayo score 1 patients, although scarce in our dataset, were similar to healthy controls, but the population centroid was pulled into quadrant 2 (Q2) due to increased contributions from stroma (CN-6) and inflamed stroma (CN-0) neighborhoods (Fig. 3F, Q2). Patients with Mayo scores 2 and 3 showed considerable overlap with one another, although patients within these groups were separated into Q3 or Q4 driven by differences in their underlying CN organization. Patients in Q3 were characterized by increased contributions from adaptive immune CNs such as lymphoid aggregates (CN-2) and B cell follicles (CN-9), whereas patients in Q4 were characterized by innate immune CNs such as the granulocyte CN (CN-3) (Fig. 3F, Q3 and Q4).

Comparing now TNFi-treated with untreated patients of the same endoscopic score (Mayo 2) highlighted changes in tissue architecture associated with TNFi treatment, even when the clinical disease severity was similar (Fig. 3G). Here, our analysis uses Mayo 2 patients due to their being evenly distributed both across current TNFi therapy and being responders to subsequent TNFi therapy. The cluster centroid for Mayo 2 TNFi-treated patients localized between Q4 and Q1 when projected onto the same principal component axes as before. This suggested that changes with TNFi treatment were predominantly associated with increased epithelial neighborhoods CN-1 and CN-8 (Q1) and decreased contributions from lymphoid aggregate (CN-2) and B cell follicle (CN-9) neighborhoods (Q3), leading to a shift toward normal-like tissue architectures upon treatment. Notably, lack of change in the granulocyte CN (CN-3, Q4) prevented Mayo 2 TNFi-treated patients from fully returning to the healthy state space (Q1). This suggested that these innate niches may be resistant to therapy, whereas adaptive immune neighborhoods normalize during TNFi treatment. Univariate analysis of TNFi-treated patients controlled for Mayo score 2 demonstrated that adaptive immune CNs lymphoid aggregate and B cell follicle (CN-2 and CN-9, respectively) showed the greatest decrease upon treatment, whereas epithelial CNs like basal epithelium (CN-8) increased (Fig. 3H). In contrast, frequencies of innate immune CNs did not change significantly upon treatment, suggesting potential treatment resistance. We observed that adaptive immune cells, contacts, and CNs were enriched in female patients compared to males (fig. S7), which may underlie the reported higher TNFi treatment response rates in females (*32*).

### TNFi response–associated cell types are enriched in female patients

**Fig. 4.**
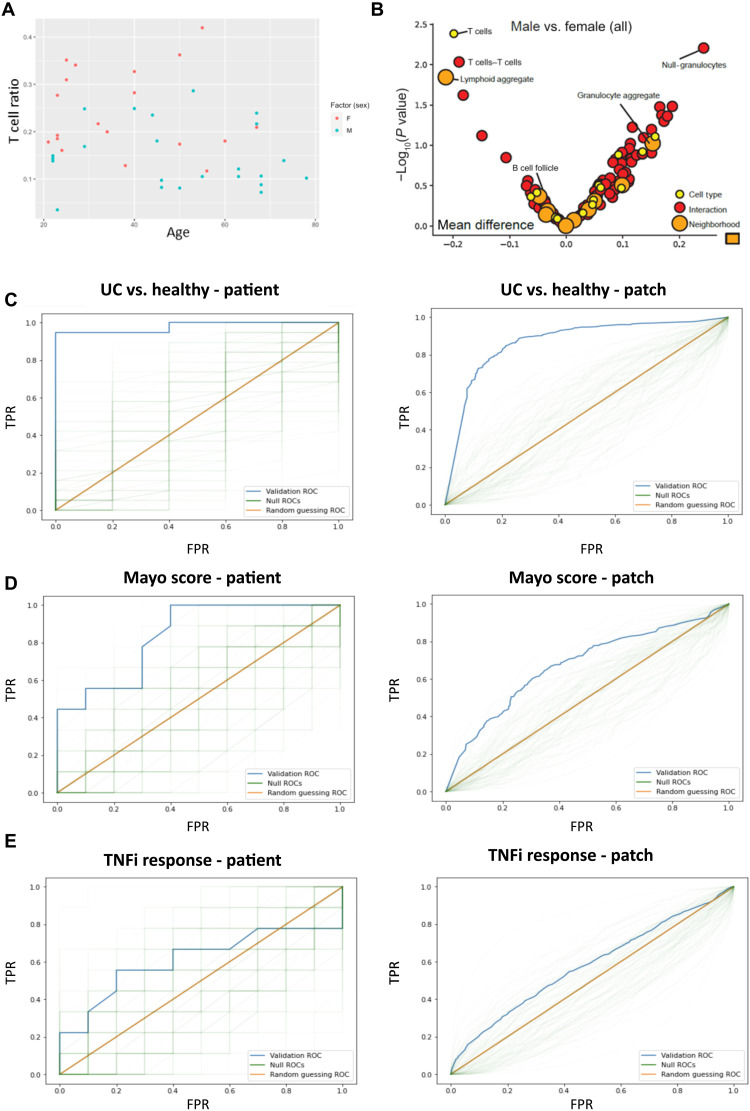
Sex and TNFi response associated architectural features. (**A**) Scatter plot of T cell ratios (no. of T cells/no. of total cells), per patient, plotted against age, demonstrates increased T cells in females (*P* < 0.01). Female patients are marked in red, and male patients are marked in blue. (**B**) Volcano plot illustrating T cell–associated interactions and neighborhoods implicated in sex differences. Dots correspond to cell types (yellow), cell-cell interactions (red), and CNs (orange). Features to the left of zero are enriched in females, and features to the right of zero are enriched in males. Features above ~1.3 are statistically significant at the *P* < 0.05 level. (**C**) Patient-level (left; *P* < 0.01) and patch-level (right; *P* < 0.01) ROC curves for UC versus healthy using patch-level training with the standard representation. The blue curve is the validation ROC, the green traces are the null ROCs, and the orange line represents the AUROC = 0.5 random guessing ROC. TPR indicates true positive rate, FPR indicates false positive rate. (**D**) Patient-level (left; *P* < 0.05) and patch-level (right; *P* < 0.05) ROC curves for mild/moderate versus severe (Mayo 1 + 2 versus 3) using patch-level training with the standard representation. (**E**) Patient-level (left; *P* = 0.26) and patch-level (right; *P* = 0.19) ROC curves for TNFi response using patch-level training with the CNN representation.

Among all patients with UC, male patients had lower T cell fractional abundance relative to female patients (*P* < 0.01). Using a multivariate regression, we still observed the dependence of T cell fractional abundance on sex in the presence of two confounding variables, age (Fig. 4A) and disease severity, that were either imbalanced between males and females in our cohort or displayed clear links to T cell ratios, respectively. For the expanded multivariate regression of T cell ratios on sex, age, and Mayo, the coefficient on sex was still significant (*P* < 0.05) and was consistent with increased T cell ratios in females across age groups. A closer examination of T cell subsets (Fig. 4B) revealed that the sex-associated T cell populations were those associated with T cells in lymphoid aggregate CNs, rather than those in CNs associated with follicles or epithelial layers. Consistent with our observations regarding T cells being a primary responder to TNFi therapy, our patient cohort had higher rates of TNFi response among female patients than male patients, although the difference missed the standard threshold for statistical significance (*P* < 0.055). Although we identify the clear difference in T cell count between males and females, Fig. 4 (A and B) also highlights the difficulties of the more clinically relevant patient-level prediction (such as predicting sex from T cell count) even when the input variables have statistically significant differences between experimental groups. Nonetheless, with T cell subsets potentially associated with response to TNFi therapy, we next set out to determine whether we could predict TNFi response. These predictions are assessed at both the patch level and the patient level as well as using both images and tabular datasets for inputs.

### Training and classification are performed at both the patch and patient level

We trained our model using two approaches. In the first approach (patch-level training), we input patch-level representations into our model, which, in turn, output patch-level predictions. The patch-level predictions were then averaged to give a patient-level prediction. In the second approach (patient-level training), we aggregated the patch-level representations into a patient-level representation. This patient-level representation is input into the model, which outputs a patient-level prediction. These two approaches address potential complications associated with patch-level predictions: In situations where the signal is only present in a few patches, the majority of patch-level embeddings will effectively be incorrectly labeled; in contrast, when the signal is dispersed across many patches, aggregating the patch-level embeddings into a patient-level embedding may unnecessarily blend together distinct and salient samples (fig. S8). When performing patient-level classifications with patch-level training, we average patch-level predictions to get patient-level predictions. While we additionally tried the maximum and the average of the top and bottom 5, 10, or 20% of the patch-level predictions, we did not observe any improvement in results.

Our reported results are from a leave-one-out cross-validation, also known as *n*-fold cross-validation, in which we produce predictions for a given patient “*i*” from an L2-regularized logistic regression trained on all the remaining patients. We evaluated performance based on both the patient-level validation accuracy and receiver operating characteristic (ROC) curve. We classified patients from their patient-level prediction, where the classification threshold for a given patient *i*, with *i* ranging over all patients, is the maximizer of the Youden index for patient-level classification in a further nested leave-one-out cross-validation, which uses all patients other than patient *i*. The L2 penalty is determined by selecting one of the four regularization strengths over a 4-log range, which maximizes validation area under the ROC (AUROC). For comparison, we repeat the analysis *N* = 100 times after randomly permuting the response at the patient level. We present the resulting validation ROC curves, which we dub “null ROC curves,” and *P* values, which are valid for testing the null hypothesis that the predictors and response are independent. We also tried an L1-regularized regression when using the standard representation but did not observe any improvement in results.

### TNFi response prediction is performed using two different image representations

To predict TNFi response in patients, we first addressed uncertainties in the relevant size scale of biological features by considering two different featurizations of our data. Our first representation (standard representation) used cell ratios, cell-cell interaction ratios, and CN ratios, as previously reported (*28*). Our second representation [convolutional neural network (CNN) representation] is the output of the last hidden layer of ResNet-50, a standard CNN architecture pretrained on ImageNet, using 1000 × 1000 pixels Voronoi patches resized to 500 × 500 pixel images as inputs. By inputting the Voronoi images into ResNet-50 for feature extraction, we avoid user assumptions regarding relevant features. We evaluated the consistency of our results with respect to different choices of pretrained ImageNet architectures (ResNet-18, ResNet-34, ResNet-101, ResNet-152, and ShuffleNet v2) and input patch sizes (500 × 500 pixels resized to 224 × 224 pixels).

For a given binary classification task, we input these representations into a model. The model, in turn, outputted a “prediction.” Here, the prediction is a value between 0 and 1 and is informally interpreted as the probability that the model believes the input belongs to class 1 versus class 0. We performed an analysis of patch- versus patient-level training and classification before predicting TNFi response (see the Supplementary Materials). We further assessed the accuracy and stability of our neural networks by predicting UC versus healthy as well as disease severity (Fig. 4, C and D; see the Supplementary Materials).

### Benchmarking predictive neural networks reveals that they are accurate and stable

Before predicting TNFi response, we benchmarked our approach using two binary prediction tasks of increasing difficulty: UC versus healthy (*n* = 19 and 5) and severe (Mayo 3, *n* = 9) versus mild/moderate (Mayo 1/2, *n* = 10). The first task, UC versus healthy, was readily determined by examining the biopsy’s gross histological features. The second, severe versus mild/moderate UC, was not readily apparent. We evaluated performance for both the standard and CNN representations using both patient-level and patch-level training and further evaluated performance stability for the CNN representation by trying different pretrained networks (ResNet-18, ResNet-152, and ShuffleNet v2) and input patch sizes (500 × 500 pixels).

We achieved a high level of success in predicting UC versus healthy (Fig. 4C). When training at the patch-level with the CNN/standard representation, we observed two/three misclassifications and achieved a validation AUROC of 0.95/0.98, respectively (table S2). Training at the patient-level with the CNN/standard representation resulted in one/three misclassifications and gave similar validation AUROCs for both. We were also successful in predicting severe versus mild/moderate UC, although this task appeared to be more difficult (Fig. 4D). For the CNN/standard representation, training at the patch-level resulted in four/seven misclassifications and gave validation AUROCs of 0.79/0.84. Training at the patient-level resulted in five/four misclassifications for the standard representation and validation AUROCs of 0.76/0.80, respectively (table S4).

When predicting UC versus healthy, our results are stable with respect to the choice of pretrained neural network and input patch size. We never had more than three misclassifications and observed similar validation AUROCs. In general, patch-level training resulted in better validation results than patient-level training (table S4). For predicting disease severity, we saw slightly more variable results depending on the choice of pretrained neural network and input patch size. We typically had four to six misclassifications when training at the patch-level , but patient-level training had consistently worse and more variable validation AUROCs (table S4).

Generally, our patch-level training outperforms and is more stable than patient-level training. At times, patch-level training also tends to result in seemingly more reasonable patient-level predictions (fig. S9). To understand why this is the case, we also examined validation patch-level ROC curves for both tasks (Fig. 4, C and D). Our success in predicting at the patch level indicates that the signal for the tasks of interest is present in a nontrivial number of patches. In principle, this is likely due to a high level of autocorrelation among patches from the same patient. However, the relative tightness of the null distribution patch-level ROC curves compared to the null distribution patient-level ROC curves suggests that patches from the same patient are not exactly identical or perfectly correlated. If patches from the same patient contain salient, heterogeneous examples of the relationship between predictors and response, then patch-level prediction would result in a less variable, more generalizable model.

### Predicting TNFi response in patients with UC is variable and unstable

With our approach validated, we next predicted TNFi response for responders (*n* = 9) versus resistors (*n* = 10). For the CNN/standard representation, training at the patch level resulted in six/seven misclassifications and gave validation AUROCs of 0.66/0.49, respectively (Fig. 4E). Training at the patient level resulted in 4/11 misclassifications and gave validation AUROCs of 0.74/0.46, respectively (table S2). While the results for the CNN representation may seem promising, the instability over different choices of pretrained ImageNet architecture and input patch size is a serious cause for concern. Varying these choices resulted in up to 9/10 misclassifications for both patient-/patch-level training and showed that the four misclassifications were clearly an anomaly (table S2). Although some patient-level validation AUROCs are reasonably large, the results varied considerably depending on the choice of pretrained architecture, getting as low as 0.48. The highest validation AUROC that we got from patient-level or patch-level training had a *P* value that missed the significance mark (*P* = 0.13) (fig. S10). We did not observe any distinguishing characteristics, including whether patient biopsies were obtained before or after treatment, with respect to which patients were correctly or incorrectly classified.

## DISCUSSION

UC and other autoimmune diseases are among the most common inflammatory disorders worldwide (*1*, *33*, *34*). Biological therapies such as TNFi have improved patient quality of life and reduced long-term complications of these chronic diseases. However, because of the toxicities, adverse effects, substantial failure rates, and high treatment costs associated with biologics, clinicians are in need of predictive biomarkers to guide the choice of therapy (*12*, *14*, *32*). Recent studies using single-cell technologies such as scRNA-seq and mass cytometry have attempted to identify such biomarkers and differences in the intramucosal immune systems of patients compared to healthy controls; however, these technologies cannot measure tissue architecture and the spatial interactions of cells (*20*, *21*, *35*). Cellular organization is known to provide critical insights into disease progression and treatment response (*28*). We therefore reasoned that analyzing the gut architecture in patients with UC, treated or not with TNFi, using highly multiplexed microscopy to interrogate the tissue at multiple levels simultaneously (i.e., at the level of cell-type frequencies, cell-cell contacts, and higher-order architectural features such as CNs) should enable previously unidentified biologic insights.

By taking advantage of CODEX’s unique highly multiplexed tissue visualization with single-cell spatial resolution, we were able to analyze the spatial relationships between different cells to achieve important insights into the underlying architectural pathophysiology of UC. The ability to map marker expression onto cell type and CNs, as in the case of elevated CD137 on neutrophils in the inflamed vasculature neighborhood, is a powerful tool for addressing how tissue microenvironment affects cell functional states. These spatial insights would not have been possible with single-cell technologies, or standard histological approaches, alone. We used semiautonomous algorithms to define and map cell types, cell-cell contacts, and neighborhoods in the gut across the UC inflammatory spectrum and created a comprehensive tissue atlas of the UC gut. We created a cloud-based software platform to make this tissue atlas publicly available and queryable. This interface allows the scientific community to explore our dataset, generate, and test additional hypotheses. This atlas enabled the modeling of disease severity and the identification of cellular niches that respond to TNFi treatment and that differ between sexes in our patient cohort. Our results suggest an intriguing biological basis that may underlie some of the complex sex differences observed in patients with UC (*36*). These observations are consistent with existing literature identifying stronger inflammatory responses in women. However, note that the magnitude of sex-dependent incidence rates for UC in women is not as extreme as is observed in other autoinflammatory disorders (*37*). It is as yet unclear as to whether this biological basis derives from sex-associated behaviors or is directly linked to sex. We were additionally able to identify tissue architecture–dependent signals associated with TNFi resistance. Unfortunately, the outputs from our models suggest that this signal is lost amidst the complex resistance-associated patient heterogeneities. This may be improved by future advances in how cell types are classified from CODEX data and how spatial interactions are represented. However, given the current technology, the observed heterogeneity is sufficient that marked increases in sample size would be required to even begin to resolve the relevant patient populations.

Last, we examined the application of neural networks to CODEX data as a subset of the more general problem of using neural networks for image analysis in the setting of medical experiments, where having large sample sizes is often impractical. We developed a set of recommendations for reinforcing confidence in the quality of the analytical procedure and its outcomes for similar experiments, which should be useful for guiding other investigators. Critically, we strongly suggest using *k* > 1 when using *k*-fold cross-validation. Using a onefold cross-validation with such a small number of patients gives highly variable and, at times, overly optimistic results depending on which patients are held out. Other recommendations include generating sets of null ROC curves for statistical comparison with the experimental ROC curve, considering model performance and any underlying patterns in which patients are correctly or incorrectly classified and reporting both validation AUROCs and misclassification rates. In our benchmark tasks, we observe differences between our misclassification rates and the misclassification rates resulting from selecting the optimal threshold derived from the validation data. This points to the difficulty of selecting a prediction threshold from the training data that will generalize to new, unseen data (table S2). Furthermore, fine-tuning neural networks is not advisable in data regimes (small number of correlated samples) like ours. We observed that fine-tuning did not appreciably change our results and was computationally intensive to the extent that *k*-fold cross-validation along with the generation of null ROC curves would be impractical. Notably, while our neural networks suggested that much of the clinically relevant signal that we observed appears to be well dispersed throughout the tissue, the lack of significance in B cell changes (Fig. 3, B and C) likely reflects slice-slice variability with regard to the relatively large B cell follicle tissue architectures. As the CODEX technology matures, we anticipate that being able to feasibly image multiple slices per biopsy, across greatly increased numbers of patients, will positively affect the variability observed in our dataset.

This study offers insights into the central biological question of how higher-order tissue properties emerge from a cellular organization. Although we have identified architectural motifs associated with TNFi response in UC in this patient cohort, it is still unclear how these motifs mechanistically lead to TNFi resistance and how these might be therapeutically altered to regain TNFi responsiveness. The presence of these motifs provides insights into the cellular basis of inflammation, although it is still uncertain as to whether these motifs are inherently pathological or whether they are required components of a normal inflammatory response and it is only their aberrant persistence that is pathological. Here, animal models mimicking UC coupled with knockout strategies can help with understanding pathogenesis. The use of longitudinal data, with biopsies obtained before and after TNFi treatment, is critical for further experiments. Our data, to an extent, support existing literature that suggests that combination therapies targeting innate immune cells such as neutrophils merit further investigation (*38*–*40*). However, the neutrophil-associated signal observed in our study was so low as to not reach statistical significance. This discrepancy suggests that exploring different approaches to patient-level representation may be useful in predicting TNFi response. We encourage testing and validating such beliefs on new patient data. The tools that we have developed are broadly applicable for the study of other immune-driven diseases, and the potential roles of these motifs in other autoinflammatory disorders should be further studied.

## MATERIALS AND METHODS

### Patient recruitment and tissue collection

The University of California San Diego (UCSD) Inflammatory Bowel Disease Biobank was established through the Human Research Protection Programs at UCSD. Patients were identified and consented to follow Institutional Review Board protocols 131487 (UCSD). Patients aged 18 years and older who were presenting for routine colonoscopies from 2012 to 2018 without a history of IBDs were recruited as controls, and patients aged 18 years and older with a confirmed diagnosis of UC were recruited. Informed consent was obtained. Intestinal tissue biopsies were collected using standard biopsy forceps and immediately placed into cryovials on dry ice. These collections were taken from various areas of the distal colon. Samples were stored at −80°C before use. The study included 29 patients with UC and five healthy controls. Table S1 shows the patient characteristics. We acquired multiple regions from several patients’ biopsies of the distal colon, creating a total of 42 samples in our analysis and allowing for inter- and intrasample assessment of cellular and spatial heterogeneity.

### Participant cohort characteristics

Each patient’s clinical phenotype was assessed by an IBD specialist to define disease subtype, location, and phenotype based on the Montreal disease classification (*41*). In our cohort of 29 patients with diagnosed UC, 15 individuals were now on TNFi (table S1): two with mild UC (Mayo score 1), six with moderate UC (Mayo score 2), and seven with severe UC (Mayo score 3) based on prospectively scored endoscopic reports. Of the 14 individuals not on TNFi therapy at the time of biopsy, two had mild UC (Mayo score 1), seven had moderate UC (Mayo score 2), and five had severe UC (Mayo score 3). Outcomes and subsequent TNFi responses were collected. In the cohort of patients with UC not treated with TNFi at the time of biopsy, six patients were later treated with TNFi and could be identified in regard to their therapy outcome. Three were subsequent responders to TNFi, and three were subsequent nonresponders. The UC cohort on TNFi had seven nonresponders and six responders. Clinical information on TNFi response was not available for the remaining participants.

### Generation of CODEX DNA-conjugated antibodies

Purified, carrier-free monoclonal and polyclonal antibodies (table S2) were conjugated to unique DNA oligonucleotides (TriLink BioTechnologies) as described before (*28*). Each antibody was concentrated on a preblocked 50-kDa centrifugal filter column (Amicon Ultra, EMD Millipore, no. UFC505096), and a partial antibody reduction was performed using a Tris(2-carboxyethyl)phosphine hydrochloride *(TCEP)*reduction solution containing 2.5 mM TCEP (Sigma-Aldrich, no. C4706-10G) and 2.5 mM EDTA (Sigma-Aldrich, no. 93302) in phosphate-buffered saline (PBS; pH 7.0). The partial reduction was allowed to run for 30 min. Toluene-deprotected, lyophilized, and maleimide-modified DNA oligonucleotides were then conjugated to the antibodies at a 2:1 w/w ratio for 2 hours, with at least 100 μg of antibody per reaction. Conjugated antibodies were washed and eluted in PBS-based antibody stabilizer (Thermo Fisher Scientific, no. NC0436689) containing 500 mM NaCl, 5 mM EDTA, and 0.1% (w/v) NaN_3_ (Sigma-Aldrich, no. S8032).

### CODEX antibody validation, titration, and staining

DNA-conjugated antibodies were validated on fresh-frozen tonsil and colon biopsy tissues under the supervision of a board-certified surgical pathologist (C.M.S.), and staining patterns were confirmed with online databases [The Human Protein Atlas, www.proteinatlas.org (*21*); Pathology Outlines, www.pathologyoutlines.com] and the published literature. Flash-frozen colon biopsies from patients with UC and healthy controls were embedded into a precooled (4°C) optimal cutting temperature medium (VWR/Sakura, no. 25680-930) in a cryostat and refrozen immediately. Five to seven biopsies were assembled into tissue arrays, sectioned at 7 μm, and placed on 22 mm–by–22 mm glass coverslips (Electron Microscopy Sciences, no. 72204-01) precoated with poly-l-lysine (Sigma-Aldrich, no. P8920). Coverslips were stored at −80°C until further use. CODEX staining buffers S1, S2, S4, and H2; blocking buffer; rendering buffer; and stripping buffer were prepared as described before (*28*). Coverslips were thawed to room temperature on Drierite indicating desiccant (Thermo Fisher Scientific, no. 07-578-3A) for 2 min, incubated in 100% acetone at room temperature for 10 min, and air-dried on top of a humidity chamber for 2 min. Then, tissues were rehydrated in buffer S1 twice for 2 min, followed by fixation in buffer S1 containing 1.6% (v/v) paraformaldehyde (PFA; Thermo Fisher Scientific, no. 50-980-487) for 10 min. Coverslips were briefly washed in S1 twice and equilibrated in buffer S2 for up to 30 min. Tissues were stained with the CODEX antibody cocktail in a final volume of 200 μl of blocking buffer per coverslip for 1.5 to 3 hours at room temperature in a humidity chamber with gentle shaking. After staining, coverslips were washed in buffer S2 twice for 2 min, fixed in buffer S4 containing 1.6% (v/v) PFA for 10 min, briefly washed in PBS three times, and incubated in ice-cold methanol for 5 min on ice. After briefly washing in PBS three times, coverslips were fixed in BS3 (3 mg/ml; Thermo Fisher Scientific, no. 21580) for 20 min at room temperature, followed by briefly washing in PBS three times, and stored in buffer S4 at 4°C.

### CODEX multicycle reaction and image acquisition

CODEX multicycle reactions and image acquisition were performed as described before (*28*). Stained coverslips were mounted onto custom-made acrylic plates (Bayview Plastic Solutions) using coverslip mounting gaskets (Qintay, no. TMG-22), and the tissue was stained with Hoechst 33342 (1:1000; Thermo Fisher Scientific, no. 62249). Acrylic plates were mounted onto a custom-designed plate holder and secured onto the stage of a BZ-X710 inverted fluorescence microscope (Keyence). Multicycle plates were prepared by adding fluorescently labeled oligonucleotides (concentration, 400 nM) in 250 μl of buffer H2 containing Hoechst nuclear stain (1:600) and sheared salmon sperm DNA (0.5 mg/ml; Thermo Fisher Scientific, no. AM9680). The first well did not contain fluorescent oligonucleotides (“blank” cycle to determine autofluorescence for background subtraction). DRAQ5 nuclear stain (Cell Signaling Technology, no. 4084L) was added to the last well at a 1:100 dilution. For details on the order of fluorescent oligonucleotides and microscope light exposure times, see table S2. Automated image acquisition and fluidics exchange were performed using a CODEX instrument and CODEX driver software (Akoya Biosciences), according to the manufacturer’s instructions, with slight modifications. Tissue overview images were acquired manually using a CFI Plan Apo λ 2×/0.10 objective (Nikon), and automated imaging was performed using a CFI Plan Apo λ 20×/0.75 objective (Nikon). After each multicycle reaction, H&E stainings were performed according to standard pathology procedures, and tissues were reimaged in bright-field mode.

### Computational image processing

Raw TIFF image files were processed, deconvolved, and background subtracted using the CODEX Toolkit uploader and Microvolution software (Microvolution) as described before (*28*). Antibody stainings were visually assessed for each channel and cycle in each tissue region using ImageJ software (Fiji, version 2.0.0), and seven-color overlay figures for selected markers were generated. TIFF Hyperstacks were segmented on the basis of DRAQ5 nuclear stain, pixel intensities were quantified, and spatial fluorescence compensation was performed using the CODEX toolkit segmenter, which generated comma-separated value (CSV) and flow cytometry standard (FCS) files for further downstream analysis.

### Cleanup gating, unsupervised clustering, and cluster validation

FCS files were imported into CellEngine (CellEngine Sign In), and cleanup gating was performed as previously described (*27*, *28*). Briefly, nucleated cells were identified by double positivity for Hoechst and DRAQ5, and out-of-focus cells were removed by gating on *Z* planes. FCS files were then reexported and imported into Vortex (*28*) using the following settings: Numerical transformation: none. Noise threshold: no. Feature rescaling: none. Normalization: none. Merge all files into one dataset: yes. Clustering was based on 34 validated cell identification antibody markers (fig. S1A) and was performed using the following settings: Distance measure: angular distance. Clustering algorithm: X-shift (gradient assignment). Density estimate: *N* nearest neighbors (fast). The number of neighbors for density estimate (*K*): from 5 to 200, steps 40. The number of neighbors: determine automatically. The optimal cluster number was determined using the elbow point validation tool at *K* = 100. Clusters containing less than 10 cells were grouped together. Data were exported as CSV files, and clusters were manually verified and assigned to cell types by overlaying the single cells from each cluster onto the stitched fluorescent and H&E-stained tissue images in ImageJ/Fiji, based on the unique cluster identifiers and cellular *X*/*Y* position, using custom-made scripts (available at https://github.com/bmyury) (*27*). Clusters with similar morphological appearance in the tissue and similar marker expression profiles were merged, and artifacts (such as fluorescent precipitates and tissue folds) were removed, resulting in 13 final clusters. Minimal spanning trees of the clusters were generated in Vortex, based on angular distance, and were exported for each marker and patient group.

### Manual gating of functional marker–positive cell types

After cleanup gating, the frequencies of major immune cell types and their expression of functional markers (fig. S2B) were manually gated using CellEngine. The investigator was blinded to tissue sample metadata. Gating strategies were validated by plotting populations on fluorescent tissue images for relevant markers, and population statistics were exported for further analysis. Statistically significant conclusions were validated by manually examining relevant cells to ensure that conclusions represent genuine cellular signal rather than spillover signal from the segmentation process.

### Generation of Voronoi diagrams and cell-cell contact matrices

Voronoi diagrams and cell-to-cell contact matrices were created using a custom-made Java algorithm with FCS files exported from Vortex, both of which were developed in Goltsev *et al*. (*27*).

### Identification of CNs

CNs were identified as previously described (*28*). Briefly, for each cell in the tissue, a “window” of 10 cells (the center cell and its nine nearest spatial neighbors) was captured, and these windows were clustered by their compositions based on the 13 cell types identified using X-shift clustering and supervised merging/annotation. We then clustered these windows using Python’s scikit-learn implementation of MiniBatchKMeans with *k* = 10. Each cell was then allocated to the same CN as the window in which it was centered. CNs were annotated on the basis of the enrichment of cell types within them and by comparing CN Voronoi diagrams to the original fluorescent and H&E-stained tissue images.

### Sex-based inference

We used a one-sided two-sample *t* test to assess T cell ratio differences between male and female patients. We used the standard one-sided *t* test for regression coefficients to assess the statistical significance of the sex coefficient in the multivariate regression of T cell ratios on sex, age, and Mayo score. We used Fisher’s exact test to assess the statistical significance of different TNFi response frequencies between male and female patients.

## Supplementary Materials

### This PDF file includes:

Supplementary Methods Figs. S1 to S10 Tables S1 to S4 References

## Notes

View/request a protocol for this paper from *Bio-protocol*.
