# Supplementary material for: A tissue atlas of ulcerative colitis revealing evidence of sex-dependent differences in disease-driving inflammatory cell types and resistance to TNF inhibitor therapy
Source: Sci Adv. 2023 Jan 20;9(3):eadd1166. doi: 10.1126/sciadv.add1166 (PMC9858501; doi:10.1126/sciadv.add1166)
Supplement: Supplementary file 1 — Supplementary Methods Figs. S1 to S10 Tables S1 to S4 References [file sciadv.add1166_sm.pdf]

Science Advances
AAAS

# Supplementary Materials for

## A tissue atlas of ulcerative colitis revealing evidence of sex-dependent differences in disease-driving inflammatory cell types and resistance to TNF inhibitor therapy

Aaron T. Mayer *et al.*

Corresponding author: Stephan Rogalla, srogalla@stanford.edu;
Christian M. Schürch, christian.schuerch@med.uni-tuebingen.de

*Sci. Adv.* **9**, eadd1166 (2023)
DOI: 10.1126/sciadv.add1166

**This PDF file includes:**

Supplementary Methods
Figs. S1 to S10
Tables S1 to S4
References

**Supplemental Methods: Prediction**

To get patch-level featurization:

1. Standard Representation: Raster scans tissue images using 1000 by 1000 pixel patches, beginning from the upper left corner and reading across, then down. Patches on the right and bottom border that were smaller than 1000 by 1000 were retained in their smaller form. For cell-type and cell-neighborhood analyses, patches were generated from the clustered cell-identity or neighborhood-identity text files. For pairwise interactions, patches were generated from the cell-identity labeled Voronoi images. In all cases, abundance ratios were obtained by normalizing to the total number of cells (for cell identity and neighborhoods) or the total number of interactions (for cell-cell interactions).
2. CNN representation: We scan the Voronoi images right-to-left and then up-down with a stride of 500 px and extract 1000 by 1000 px patches. We then resize the patches to 224 by 224 px, the standard input size of the ImageNet dataset. We also consider using a stride of 250 px to extract 500 by 500 px patches which are resized to 224 by 224 px. The images are padded with black pixels so the height and width are divisible by the stride. Convolutional Neural Net (CNN) architectures pre-trained on ImageNet have a bias towards the center of the input image. Having a stride equal to half the patch length gives overlapping patches, but it allows for every part of the image to be roughly centered in a patch. Any patches where more than 60% of the pixels are black are discarded. Using PyTorch, we instantiate an ImageNet pre-trained ResNet-50 (or ResNet-18, or Res-Net 152, or ShuffleNet v2) model, freeze all the trainable parameters, and put the model in evaluation mode. We pass each patch through the pre-trained model and extract that last hidden layer output (just before the final fully connected layer) to use as the representation for the patch.

We use a L2 regularized logistic regression with solver='lbfgs' from scikit-learn as our model and use leave-one-out cross-validation to evaluate predictive performance. More specifically, consider the ith patient. For now, we'll refer to all patients other than patient i as "training patients". For all of our tasks, we don't believe that class prior probabilities present in our data are necessarily representative of the class prior probabilities of said task's population of interest. As such we will adjust certain patch-level or patient-level predictions so that the adjusted prediction corresponds to each class having a prior probability of 0.5 *(42)*. We outline how to produce the patient-level prediction for the ith patient for each of our training methods. Whenever training or evaluating a model, we standardize our predictors using means and standard deviations from the training data.

1. Patient-level training: we aggregate our patch-level representations into patient-level representations and input patient-level representations into our model. The model then directly outputs patient-level predictions. When using the standard representation, aggregating amounts to combine our cell types, cell neighborhoods, and cell interactions to get total counts and overall frequencies. When using the CNN representation, aggregating amounts to taking the mean of all the patch-level representations. We train the model on the training patients (total number of training samples = number of training patients) and then input patient i's patient-level representation to get patient i's patient-level prediction. We then adjust patient i's patient-level prediction according to as discussed above *(42)*.

2. Patch-level training: we assign each patch the response of the patient it comes from. We input patch-level representations into our model and the model correspondingly outputs patch-level predictions. We perform a weighted training where we train on all the patches which come from training patients (total number of training samples = number of training patients * avg. number of patches per training patient). A patch from the jth patient is given weight inversely proportional to the total number of patches from the jth patient. We then input all the patch-level representations corresponding to patient i to get all of the patient i's patch-level predictions, adjust them as discussed above, and average them to get patient i's patient-level prediction.

The above gives us patient-level predictions for each patient. To determine the threshold t_i for prediction for the ith patient we perform a nested leave-one-out cross-validation. More specifically we repeat the above process with all patients excluding patient i. We then set t_i to be the choice of 0.00, 0.01, …, 0.99, 1.00 that maximizes Youden's Index for patient-level classification in this nested leave-one-out cross-validation, where when computing the ROC curve, we weight patients inversely proportionally to the size of the class (0/1) they belong to. If multiple choices maximize Youden's Index, we choose the median of the ones that do. We then classify patient i according to threshold t_i.

By using the patient-level predictions from the leave-one-out cross-validation to classify each patient according to the same threshold s for s=0.00, 0.01, …, 0.99, 1.00 we generate a validation patient-level ROC curve. Again, we weight patients inversely proportionally to the size of the class (0/1) they belong to. From the weighted ROC curve, we compute a weighted validation patient-level AUROC. In the case of patch-level training, we also have validation patch-level predictions, and we do the same

for our patch-level predictions to get a validation patch-level AUROC. In this case, a patch from patient i is given weight inversely proportional to the number of patches from patient i * the size of the class (0/1) patient i belongs to.

We run the above procedure for different regularization strengths, lambda. To go from lambda to the parameter C we input in sklearn we use the relation C = 1/(lambda * number of training samples). We present results for the C which gives the best validation AUROC. If multiple values for C give the same validation AUROC, we present results for the C which corresponds to the smallest regularization strength. We consider lambdas from numpy.logspace(-3, 0, 4) for patch-level training and lambdas from numpy.logspace(-1, 2, 4) for patient-level training. By searching over a small number of regularization parameters, we attempt to minimize the bias we incur by presenting results from the most favorable regularization strength.

Finally, we perform a permutation test to test the null hypothesis that the predictors and response are independent. More specifically, we repeat all of the above an additional N=100 times after randomly permuting the response at the patient level. Let k be the number of these additional iterations that have validation patient-level AUROC >= patient-level validation AUROC from our original, non-permuted run. We report p=(k+1)/(N+1), which is a valid p-value for testing the null hypothesis that the predictors and response are independent. We do the same for patch-level AUROC to get a second p-value for the same null hypothesis.

## Supplemental Figures

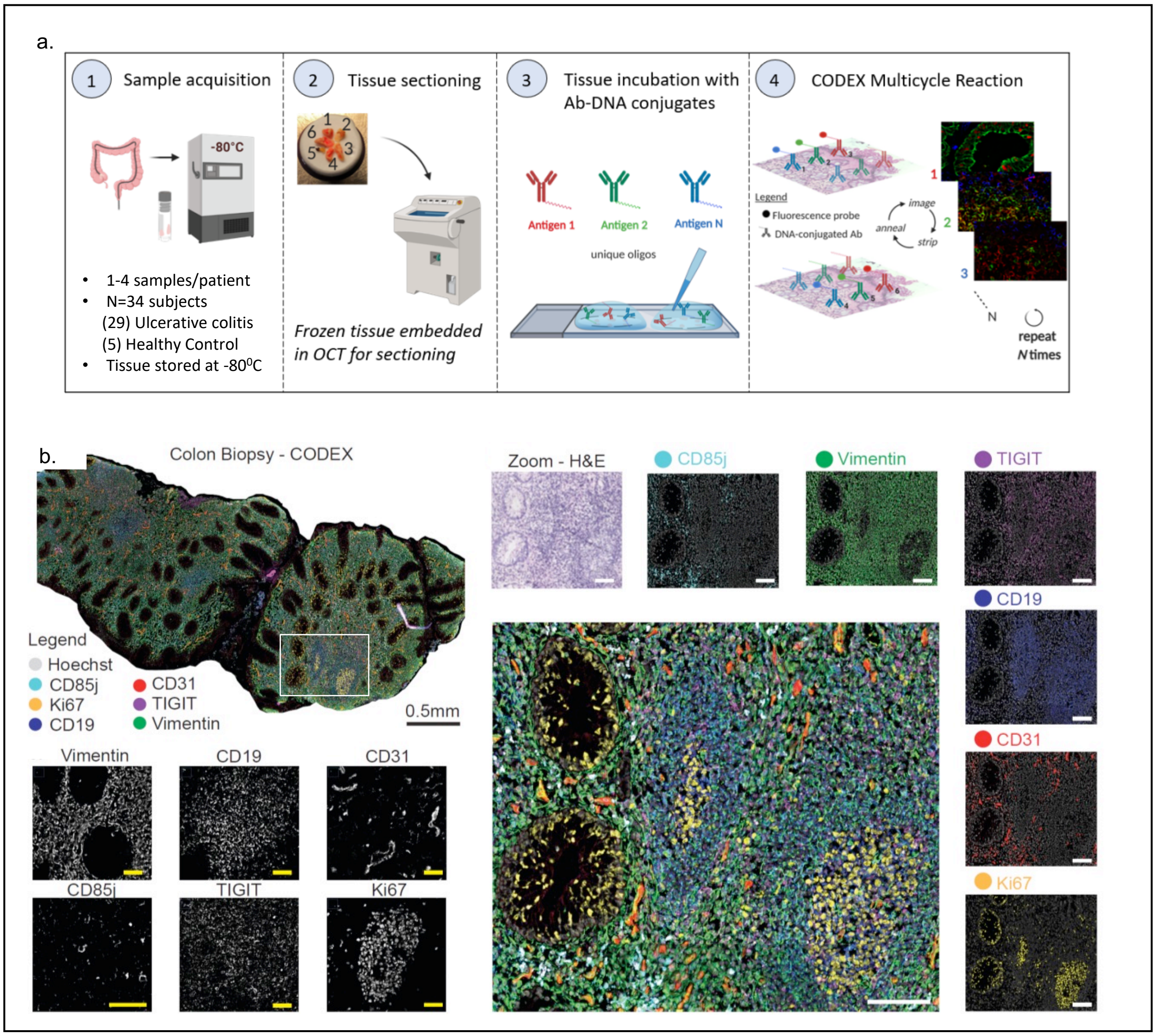


**S1a-b. CODEX imaging of tissue biomarker expression in patients with Ulcerative Colitis.**

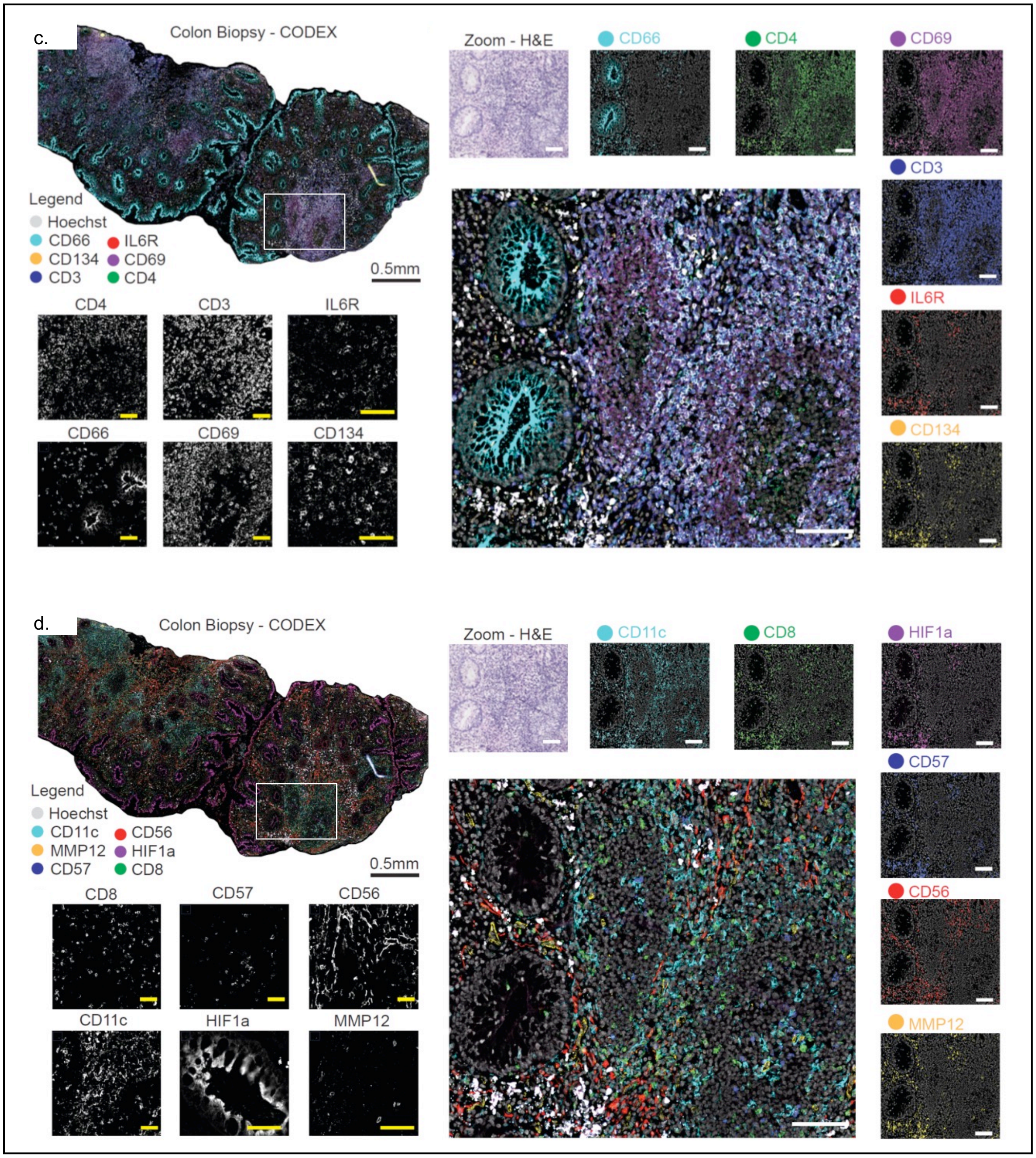


**Figure S1c-d. CODEX imaging of tissue biomarker expression in patients with Ulcerative Colitis.**

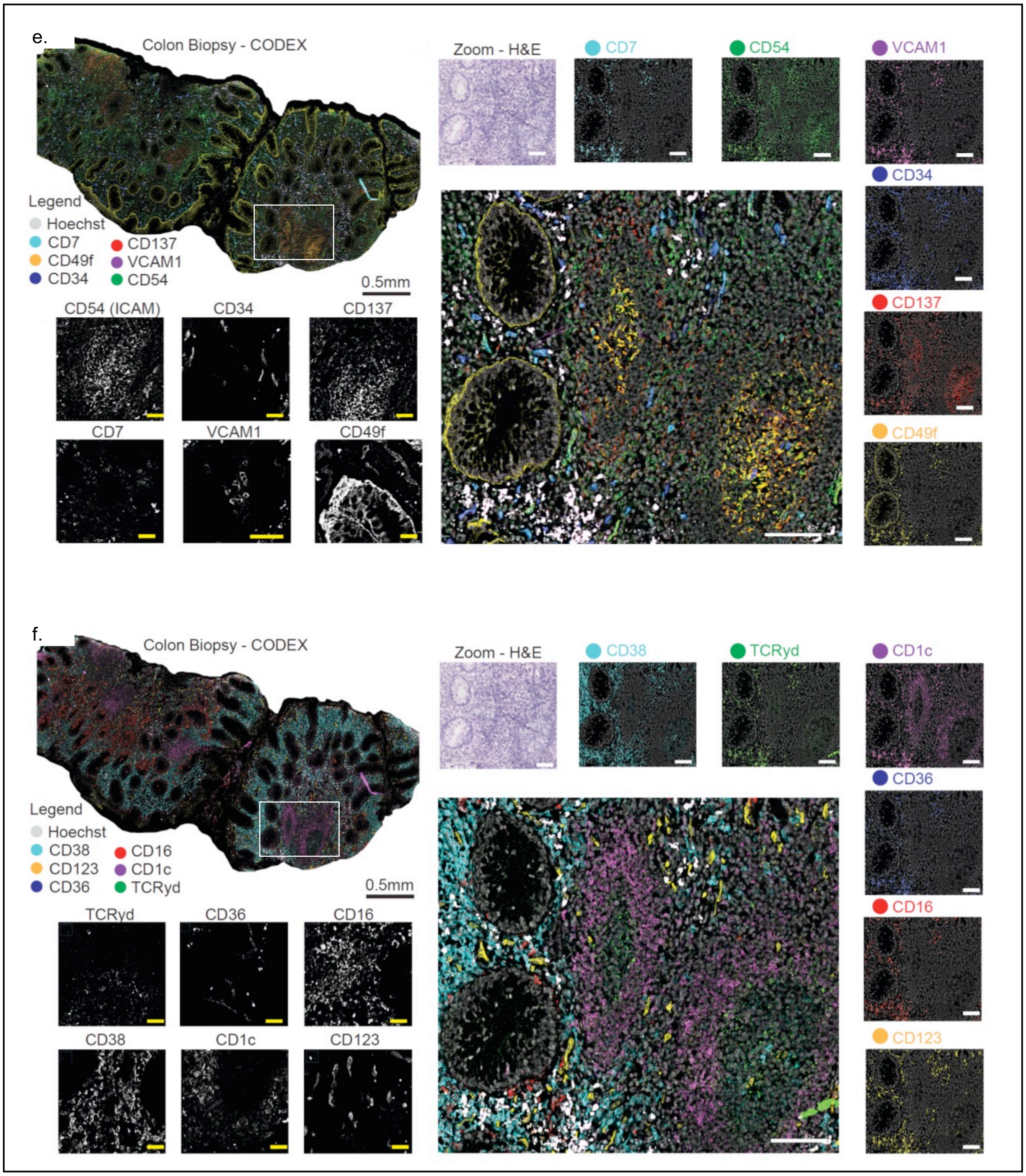


**Figure S1e-f. CODEX imaging of tissue biomarker expression in patients with Ulcerative Colitis.**

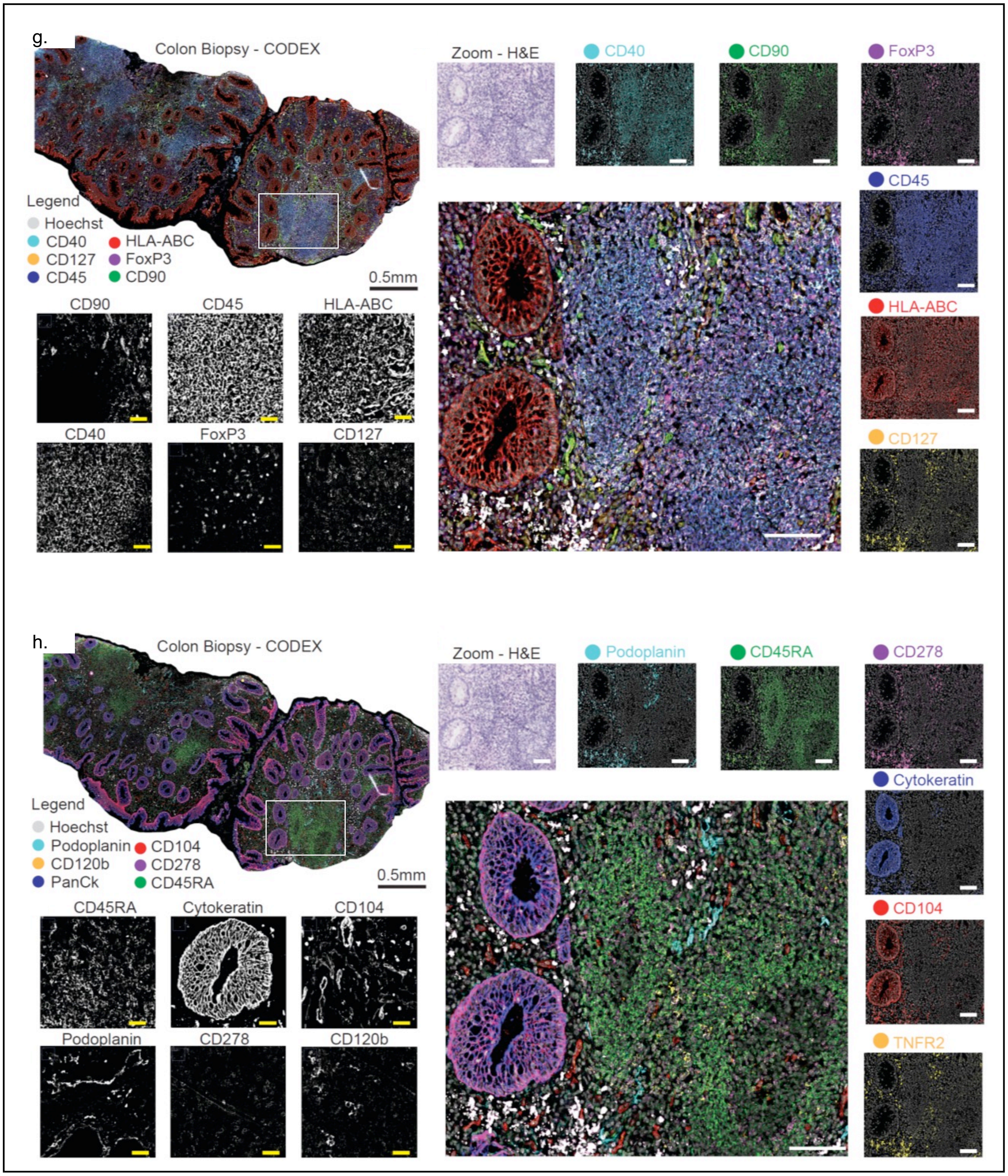


**Figure S1g-h. CODEX imaging of tissue biomarker expression in patients with Ulcerative Colitis.**

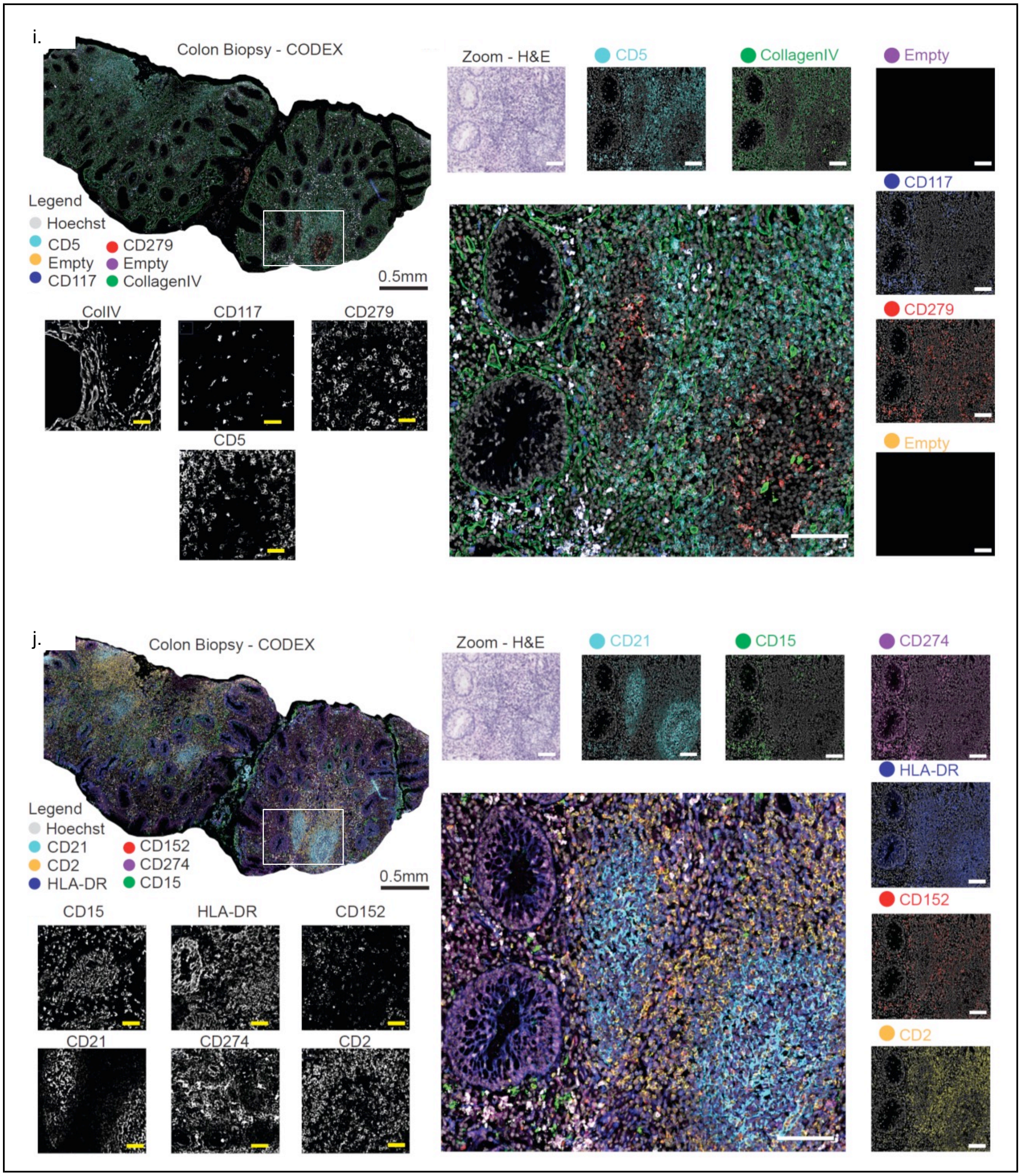


**Figure S1i-j. CODEX imaging of tissue biomarker expression in patients with Ulcerative Colitis.**

**Figure S1. CODEX imaging of tissue biomarker expression in patients with UC. (a)** Schematic overview of data acquisition process from (1) sample collection and (2) tissue sectioning to (3) tissue staining and (4) CODEX imaging. **(b)** Top left: CODEX image of a biopsy section from patient UC1 with Hoechst nuclear stain (gray), CD85j (cyan), Ki67 (yellow), CD19 (blue), CD31 (red), TIGIT (purple), and Vimentin (green) shown as a 7-color composite image selected from a total of 52 protein and 2 nuclear markers stained on the same tissue section. Bottom left: Zoomed-in view of representative staining for Vimentin, CD19, CD31, CD85j, TIGIT, and Ki67. Right: Zoomed-in view of the region denoted in the white box in panel (a) shown as a 7-color composite image (large panel) and as H&E and 2-color images of Hoechst and each of the indicated markers individually (small panels). Scale bars: yellow, 0.04 mm; white, 0.10 mm; black, 0.50 mm. **(c)** Top left: CODEX image of a biopsy section from patient UC1 with Hoechst nuclear stain (gray), CD66 (cyan), CD134 (yellow), CD3 (blue), IL6R (red), CD69 (purple), and CD4 (green) shown as a 7-color composite image selected from a total of 52 protein and 2 nuclear markers stained on the same tissue section. Bottom left: Zoomed-in view of representative staining for CD4, CD3, IL6R, CD66, CD69, and CD134. Right: Zoomed-in view of the region denoted in the white box in panel (a) shown as a 7-color composite image (large panel) and as H&E and 2-color images of Hoechst and each of the indicated markers individually (small panels). Scale bars: yellow, 0.04 mm, white, 0.10 mm; black, 0.50 mm. **(d)** Top left: CODEX image of a biopsy section from patient UC1 with Hoechst nuclear stain (gray), CD11c (cyan), MMP12 (yellow), CD57 (blue), CD56 (red), HIF1a (purple), and CD8 (green) shown as a 7-color composite image selected from a total of 52 protein and 2 nuclear markers stained on the same tissue section. Bottom left: Zoomed-in view of representative staining for CD8, CD57, CD56, CD11c, HIF1a, and MMP12. Right: Zoomed-in view of the region denoted in the white box in panel (a) shown as a 7-color composite image (large panel) and as H&E and 2-color images of Hoechst and each of the indicated markers individually (small panels). Scale bars: yellow, 0.04 mm; white, 0.10 mm; black, 0.50 mm. **(e~~l~~)** Top left: CODEX image of a biopsy section from patient UC1 with Hoechst nuclear stain (gray), CD7 (cyan), CD49f (yellow), CD34 (blue), CD137 (red), VCAM1 (purple), and CD54 (green) shown as a 7-color composite image selected from a total of 52 protein and 2 nuclear markers stained on the same tissue section. Bottom left: Zoomed-in view of representative staining for CD54, CD34, CD137, CD7, VCAM1, and CD49f. Right: Zoomed-in view of the region denoted in the white box in panel (a) shown as a 7-color composite image (large panel) and as H&E and 2-color images of Hoechst and each of the indicated markers individually (small panels). Scale bars: yellow, 0.04 mm; white, 0.10 mm; black, 0.50 mm. **(f)** Top left: CODEX image of a biopsy section from patient UC1 with Hoechst nuclear stain (gray), CD38 (cyan), CD123 (yellow), CD36 (blue), CD16 (red), CD1c (purple), and TCRyd (green) shown as a 7-color composite image selected from a total of 52 protein and 2 nuclear markers stained on the same tissue section. Bottom left: Zoomed-in view of representative staining for TCRyd, CD36, CD16, CD38, CD1c, and CD123. Right: Zoomed-in view of the region denoted in the white box in panel (a) shown as a 7-color composite image (large panel) and as H&E and 2-color images of Hoechst and each of the indicated markers individually (small panels). Scale bars: yellow, 0.04 mm; white, 0.10 mm; black, 0.50 mm. **(g)** Top left: CODEX image of a biopsy section from patient UC1 with Hoechst nuclear stain (gray), CD40 (cyan), CD127 (yellow), CD45 (blue), HLA-ABC (red), FoxP3 (purple), and CD90 (green) shown as a 7-color composite image selected from a total of 52 protein and 2 nuclear markers stained on the same tissue section. Bottom left: Zoomed-in view of representative staining for CD90, CD45, HLA-ABC, CD40, FoxP3, and CD127. Right: Zoomed-in view of the region denoted in the white box in panel (a) shown as a 7-color composite image (large panel)

and as H&E and 2-color images of Hoechst and each of the indicated markers individually (small panels). Scale bars: yellow, 0.04,mm; white, 0.10 mm; black, 0.50 mm. **(h)** Top left: CODEX image of a biopsy section from patient UC1 with Hoechst nuclear stain (gray), Podoplanin (cyan), CD120b (yellow), PanCk (blue), CD104 (red), CD278 (purple), and CD45RA (green) shown as a 7-color composite image selected from a total of 52 protein and 2 nuclear markers stained on the same tissue section. Bottom left: Zoomed-in view of representative staining for Cd45RA, Cytokeratin, CD104, Podoplanin, CD278, and CD120b. Right: Zoomed-in view of the region denoted in the white box in panel (a) shown as a 7-color composite image (large panel) and as H&E and 2-color images of Hoechst and each of the indicated markers individually (small panels). Scale bars: yellow, 0.04 mm; white, 0.10 mm; black, 0.50 mm. **(i)** Top left: CODEX image of a biopsy section from patient UC1 with Hoechst nuclear stain (gray), CD5 (cyan), Empty (yellow), CD117 (blue), CD279 (red), Empty (purple), and CollagenIV (green) shown as a 7-color composite image selected from a total of 52 protein and 2 nuclear markers stained on the same tissue section. Bottom left: Zoomed-in view of representative staining for Collagen IV, CD117, CD279, and CD5. Right: Zoomed-in view of the region denoted in the white box in panel (a) shown as a 7-color composite image (large panel) and as H&E and 2-color images of Hoechst and each of the indicated markers individually (small panels). Scale bars: yellow, 0.04 mm; white, 0.10 mm; black, 0.50 mm. **(j)** Top left: CODEX image of a biopsy section from patient UC1 with Hoechst nuclear stain (gray), CD21 (cyan), CD2 (yellow), HLA-DR (blue), CD152 (red), CD274 (purple), and CD15 (green) shown as a 7-color composite image selected from a total of 52 protein and 2 nuclear markers stained on the same tissue section. Bottom left: Zoomed-in view of representative staining for CD15, HLA-DR, CD152, CD21, CD274, and CD2. Right: Zoomed-in view of the region denoted in the white box in panel (a) shown as a 7-color composite image (large panel) and as H&E and 2-color images of Hoechst and each of the indicated markers individually (small panels). Scale bars: yellow, 0.04 mm; white, 0.10 mm; black, 0.50 mm.

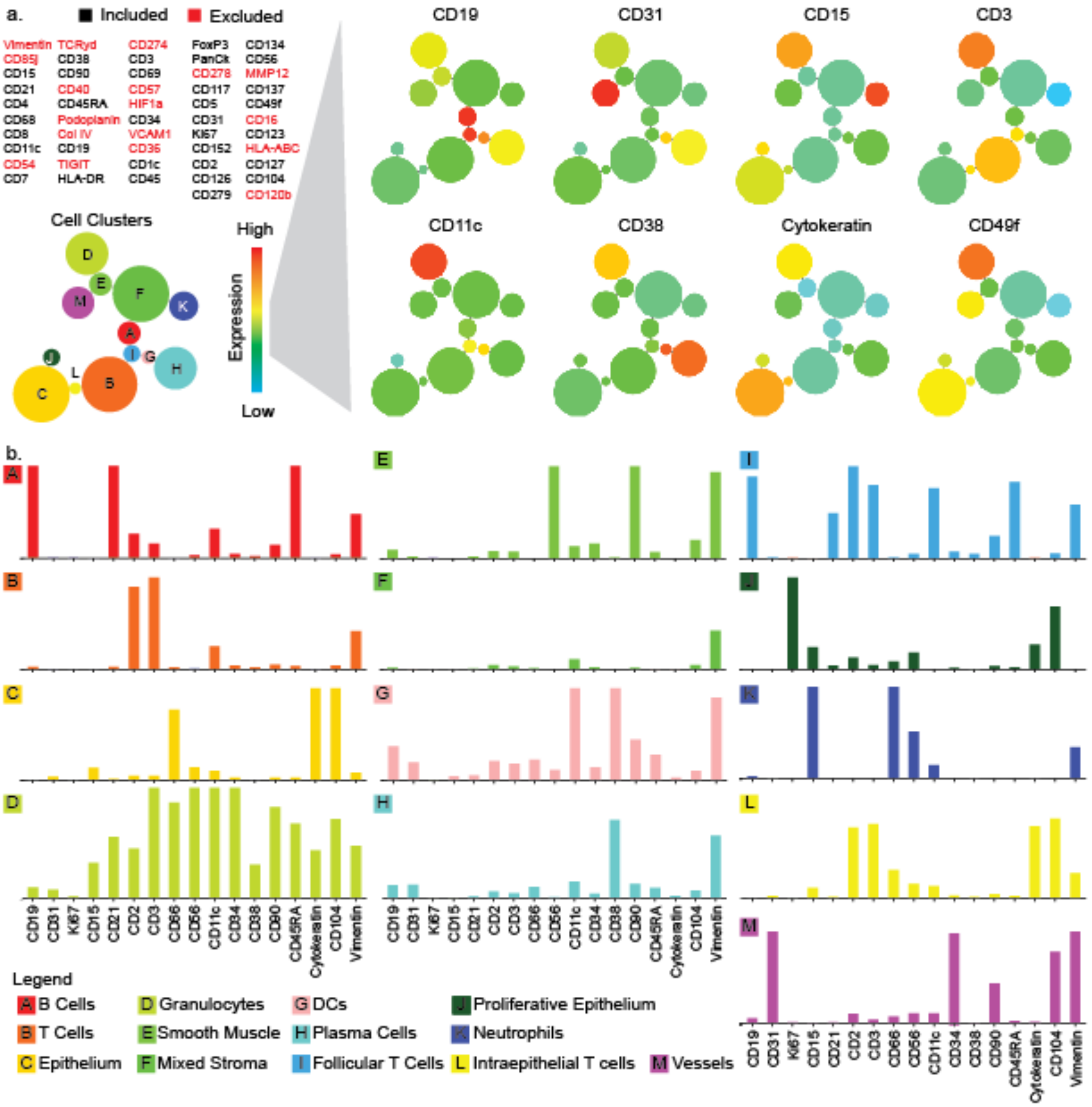

**Figure S2. Biomarker profiles for VorteX cell clusters. (a)** Minimum spanning tree representations of cell clusters. Cell types were identified in VorteX based on k-means clustering of cell-marker-intensity profiles, using the markers indicated in black. Markers in red were excluded from cluster-based identification. The size of each node represents the relative abundance of the corresponding cell type. The color of each node, from blue (lowest) to red (highest), represents the relative expression level of each marker. **(b)** Bar plots depicting min-max normalized marker intensities for each cluster. Markers that did not substantially impact clustering and some “redundant” markers (markers that strongly correlate with other markers) were excluded for visualization purposes only.

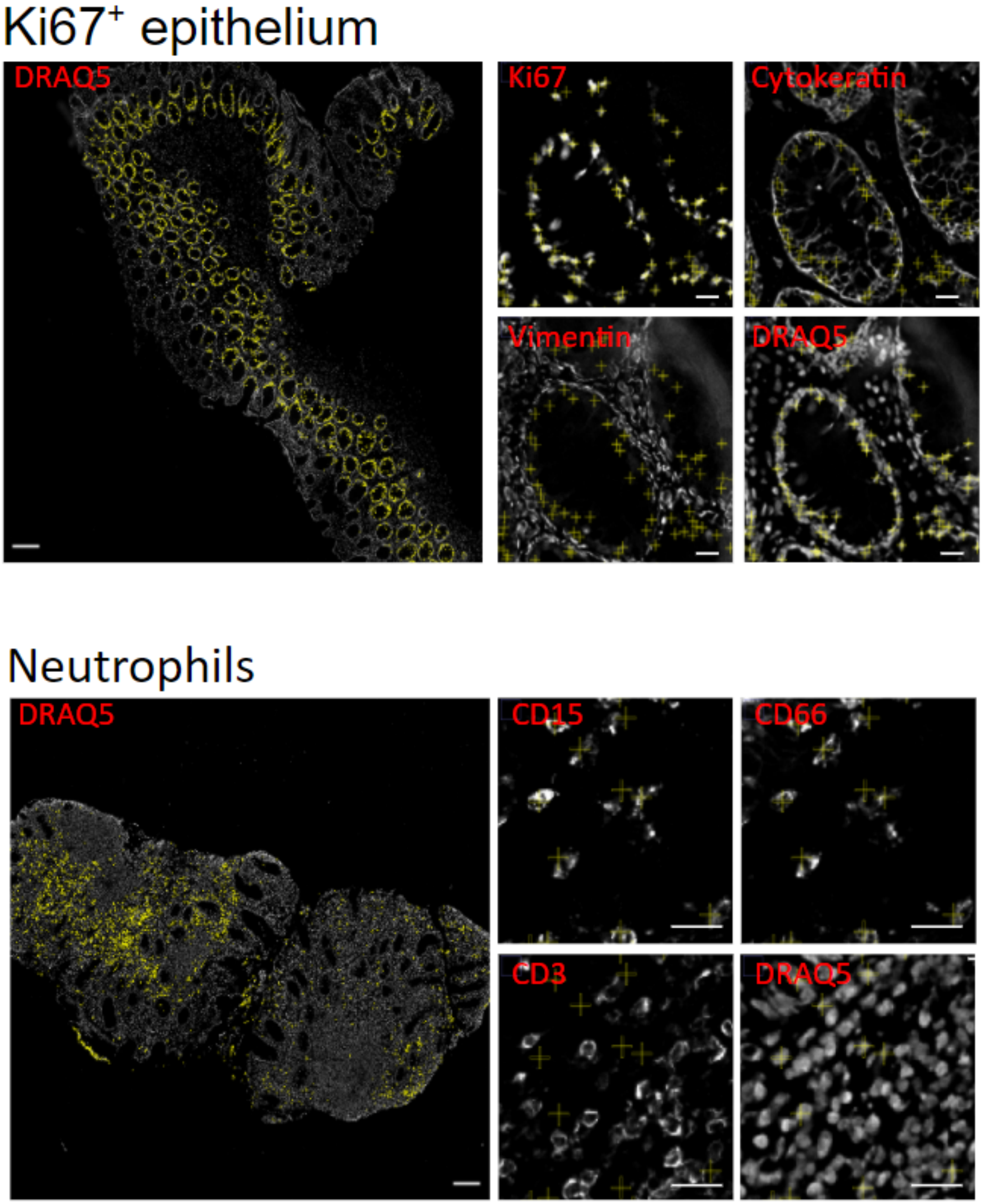


**Figure S3a-b. Cell cluster mapping.**

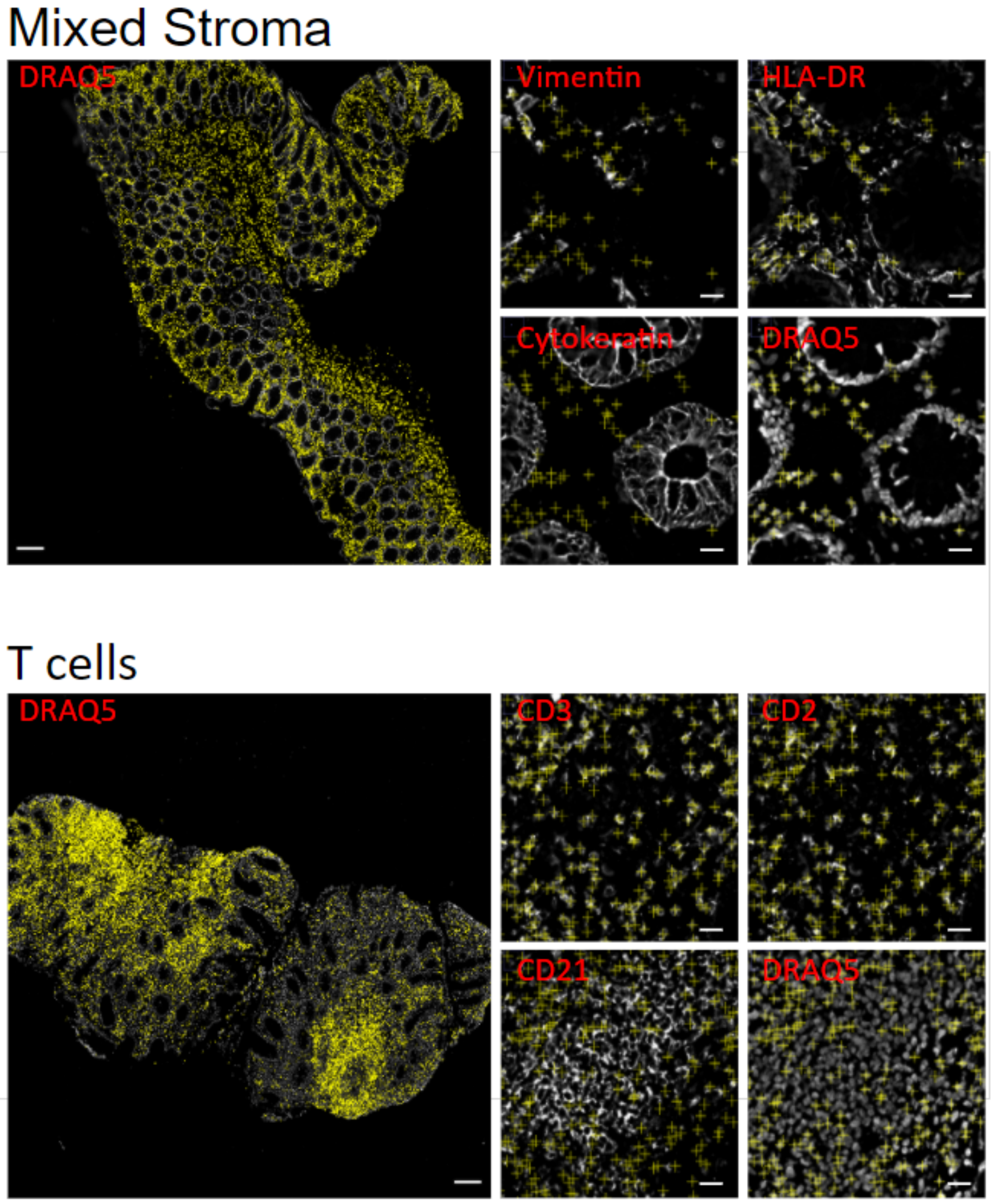


**Figure S3c-d. Cell cluster mapping.**

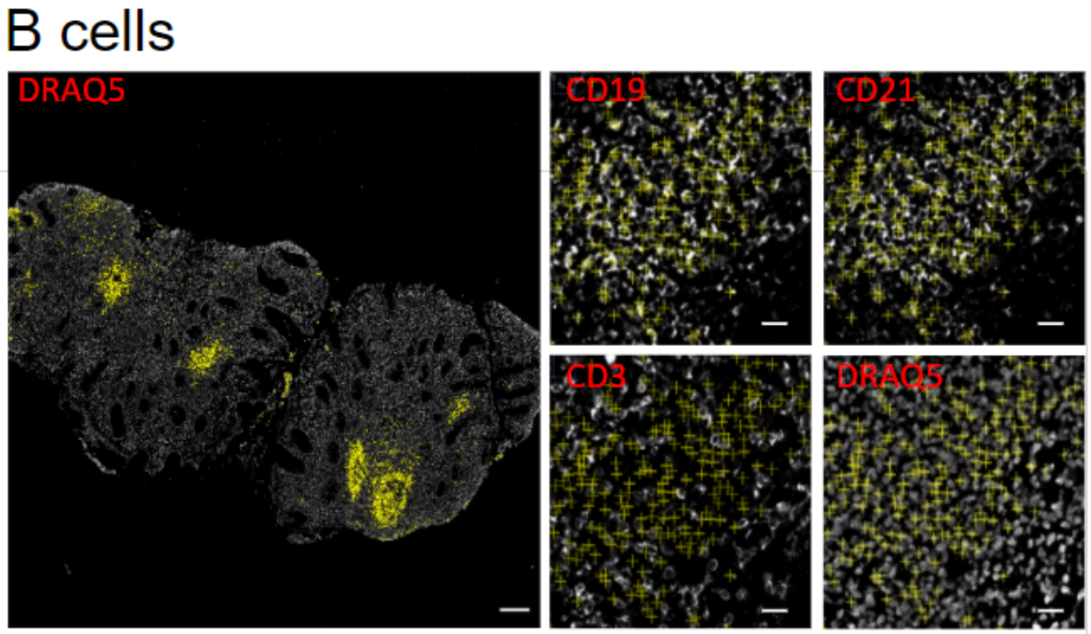


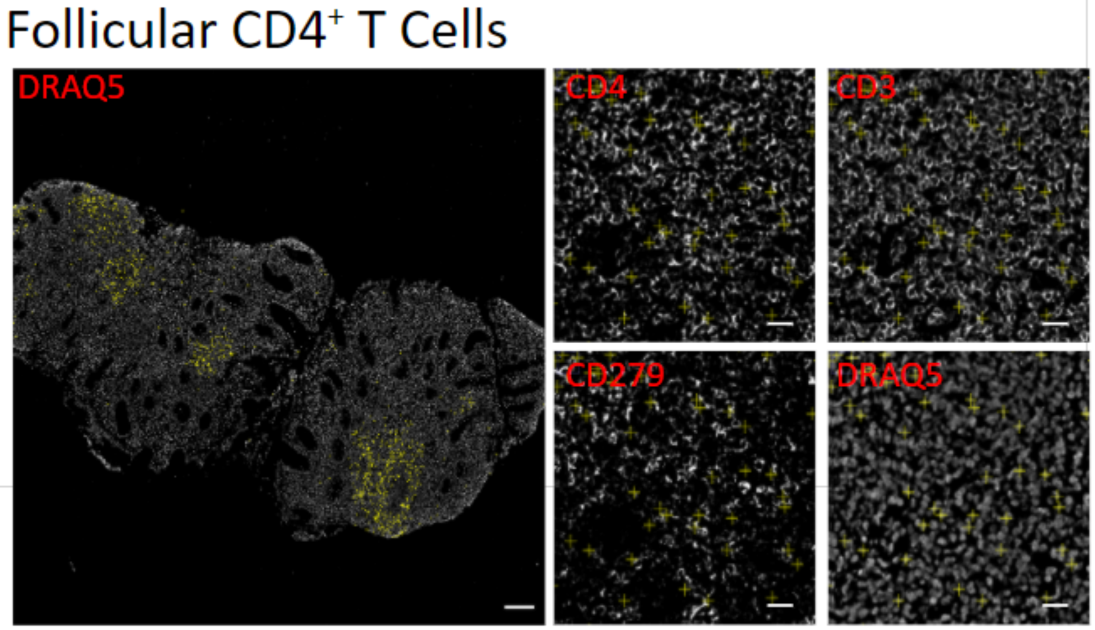


**Figure S3e-f. Cell cluster mapping.**

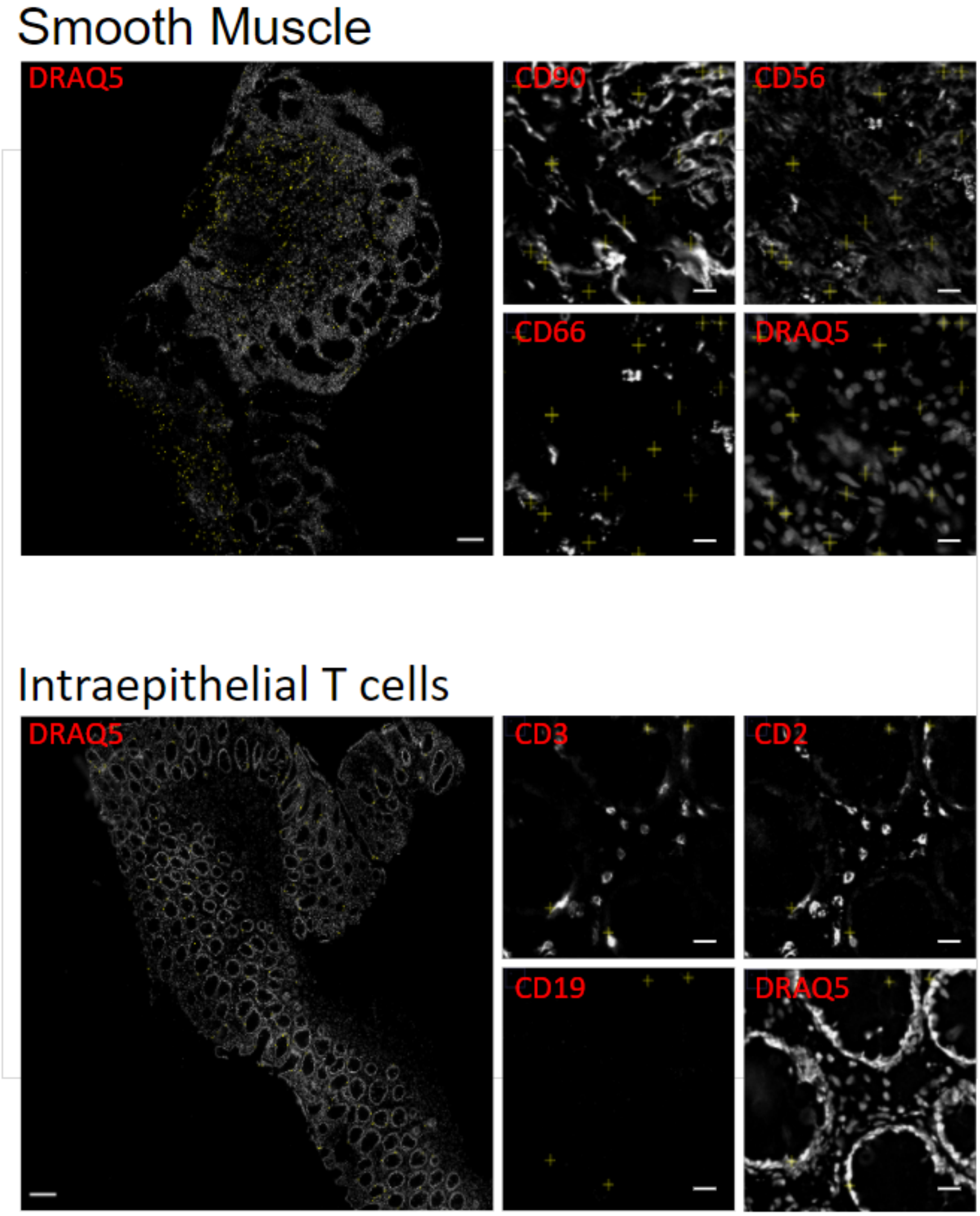


**Figure S3g-h. Cell cluster mapping.**

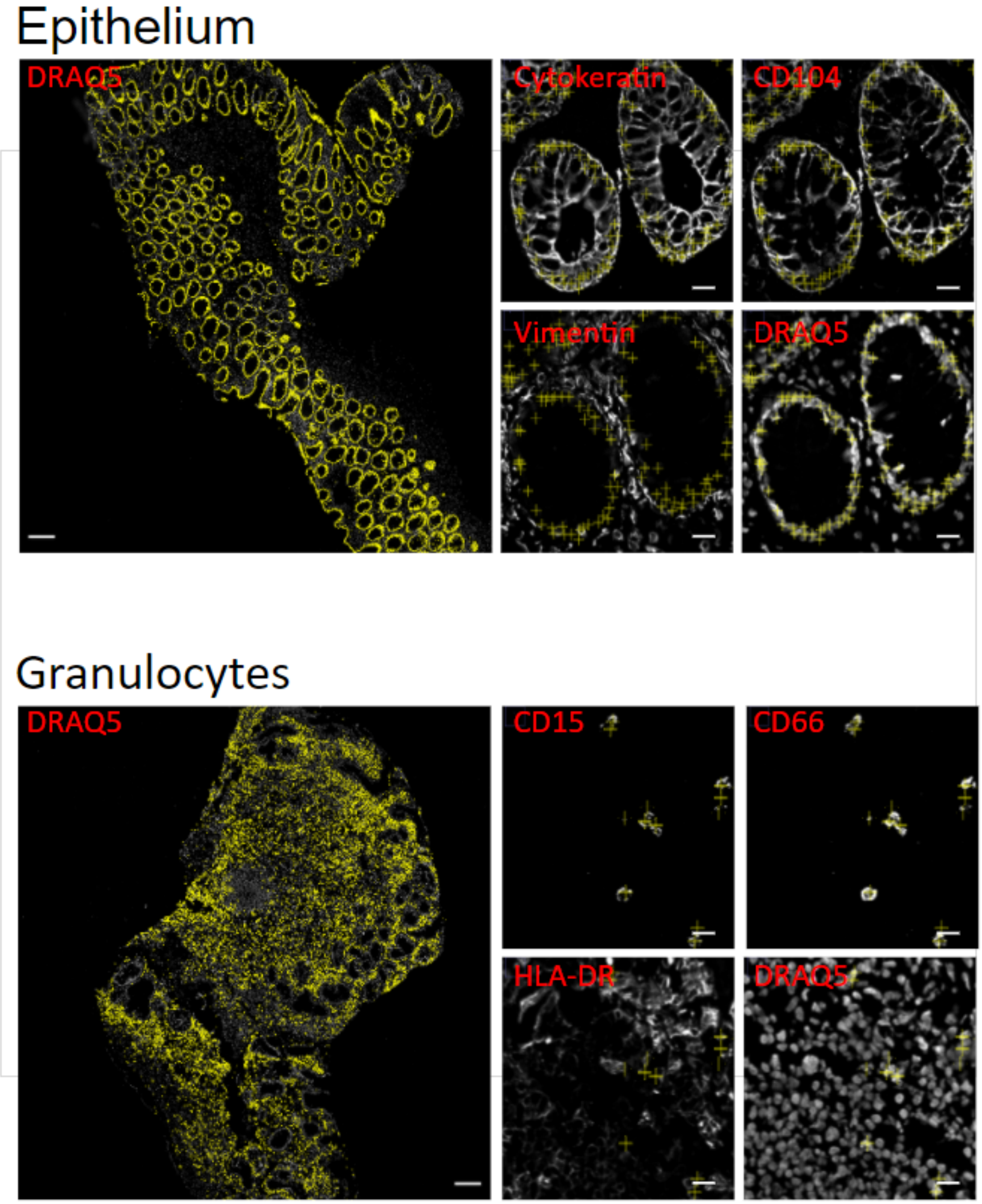


**Figure S3i-j. Cell cluster mapping**

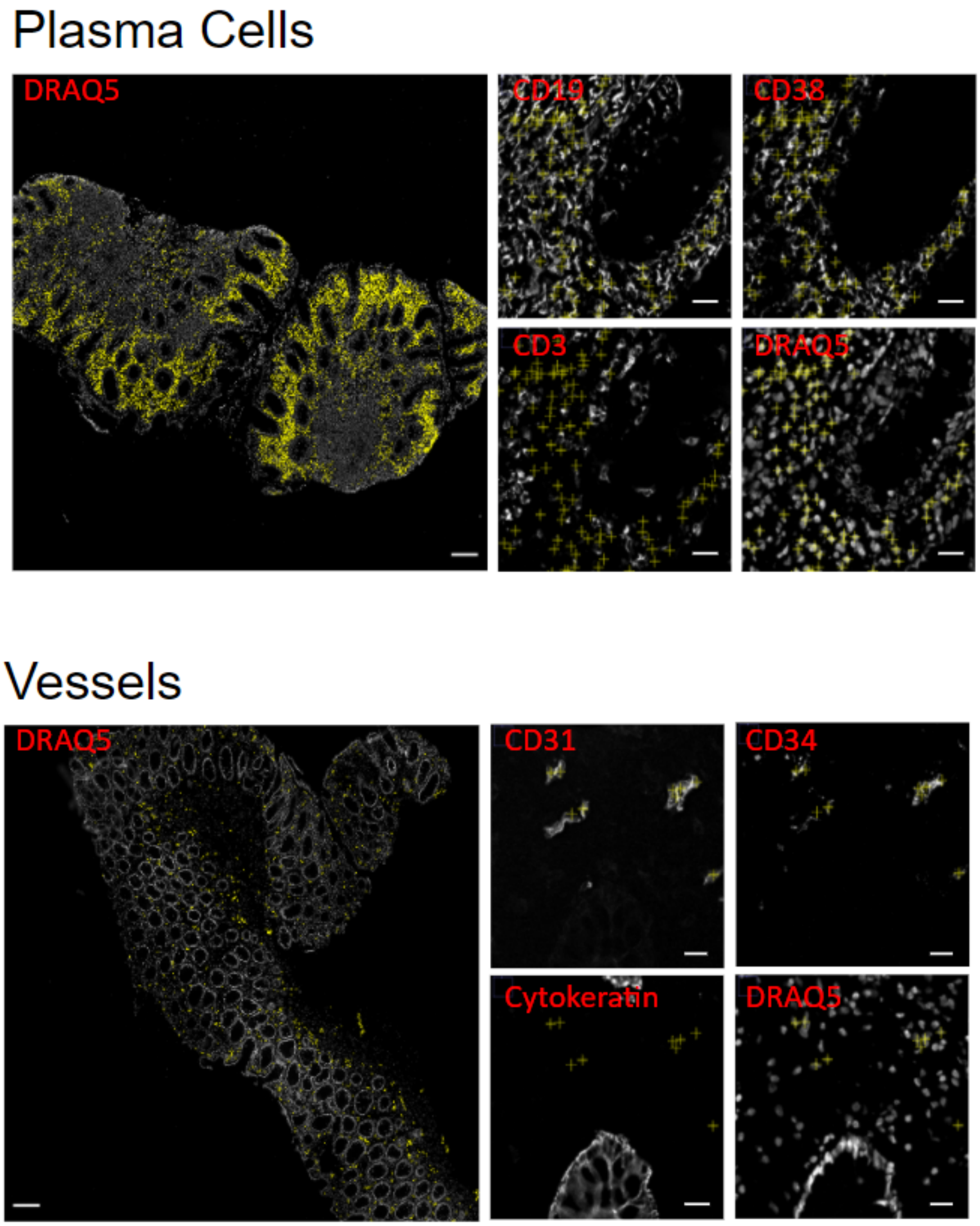


**Figure S3k-l. Cell cluster mapping.**

**Figure S3. Cell cluster mapping.** Representative images depicting the indicated cell cluster for each panel mapped to the tissue (yellow plus sign). Left: DRAQ5 nuclear staining for the whole tissue specimen with the indicated cell cluster overlaid (scale bar 200 µm). Right: Zoomed-in images of representative biomarkers that either co-localize or counter-localize with the overlaid cell cluster (scale bar 20 µm).

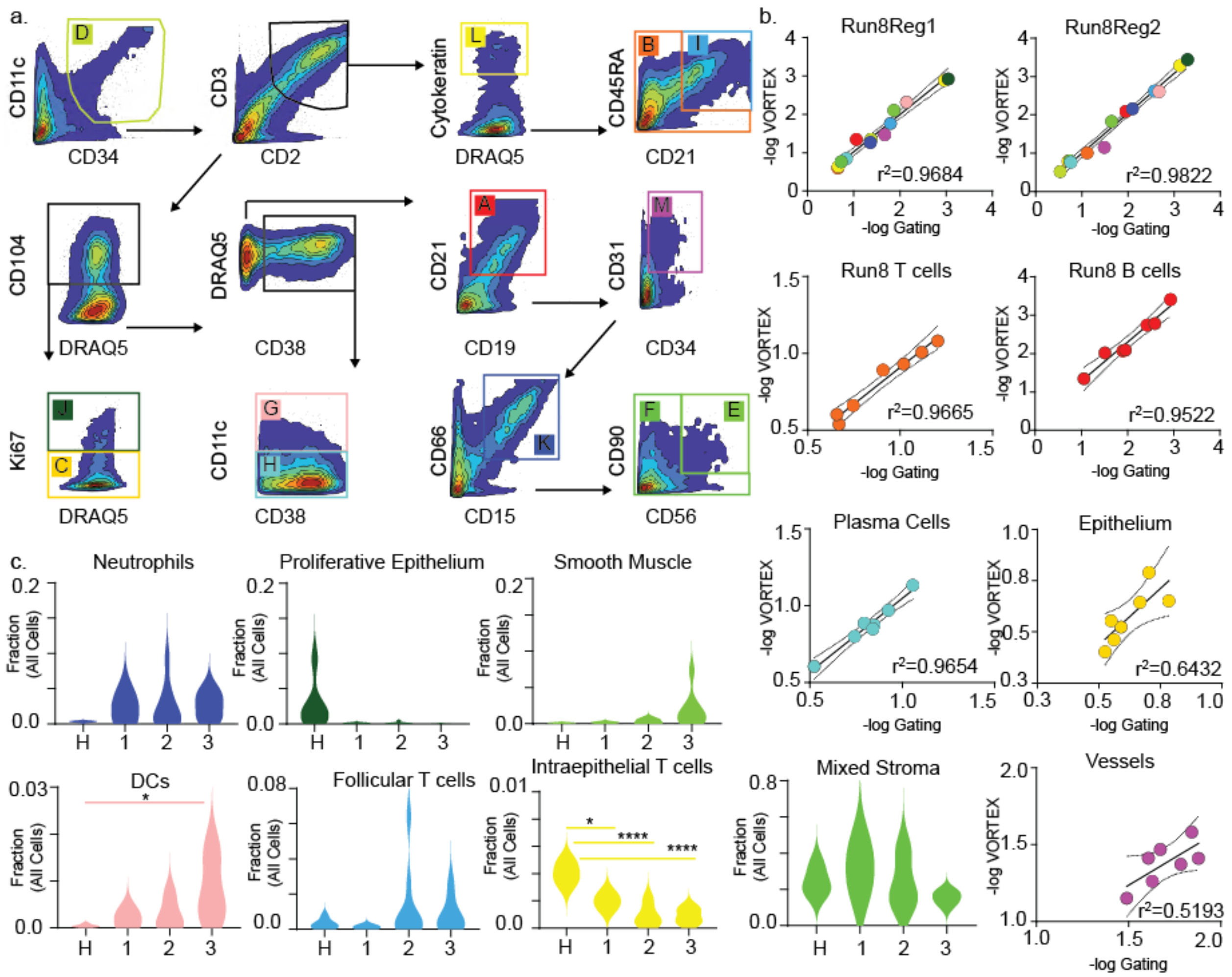


**Figure S4. Manual gating of cell types and correlation with Vortex clustering. (a)** Representative manual gating schematic. Manual gating of cell types was performed for each tissue section based on canonical biomarker expression. Visual confirmation of gated populations was performed by mapping these populations to their corresponding CODEX image as described in Figure S3 for VorteX clusters. Manual cell gating was performed blind to the VorteX clustering-based classifications. **(b)** Correlation between VorteX and manual gating for all cell types within a sample (top panel), or for each cell type across multiple samples (bottom). In general we observed good correlation between semi-autonomous (VorteX) identification and manual identification of cell types. **(c)** Violin plots indicating cellular abundance, by cell type, as a fraction of total cell numbers. Tissue samples are binned within each Mayo Score classification (H=healthy, 1= Mayo 1, 2=Mayo 2, 3=Mayo 3).

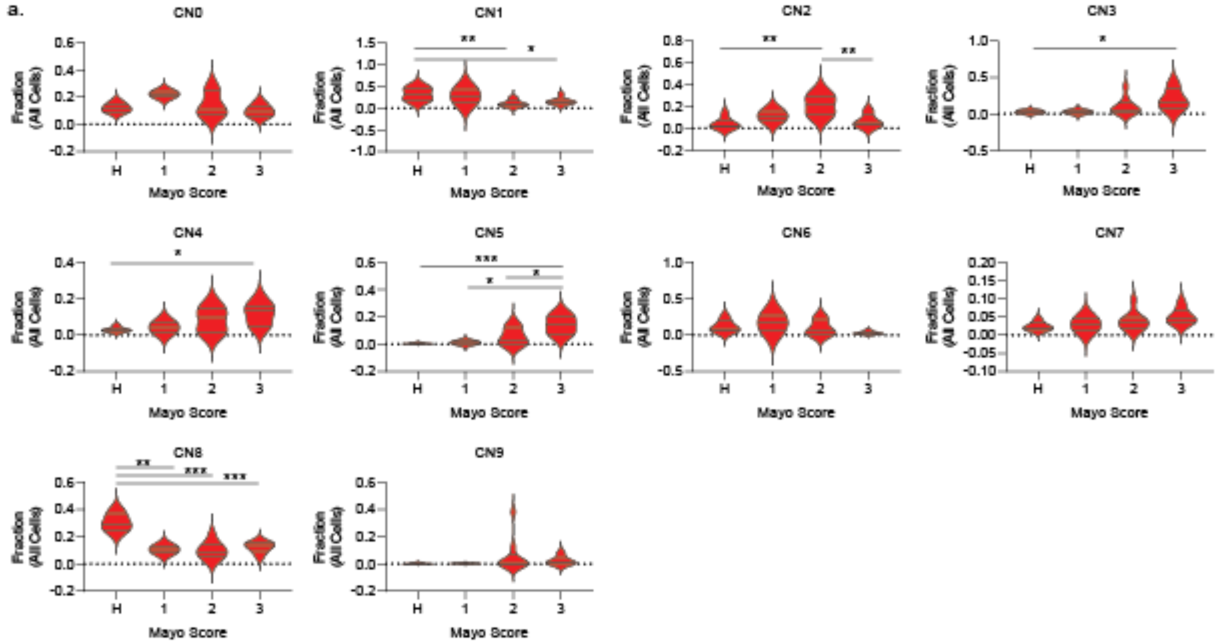


**Figure S5. Cellular neighborhoods versus clinical Mayo Scores.** Violin plots depicting the fraction of cells in the indicated cellular neighborhood versus Mayo Score. Tukey's multiple comparisons test. * p<0.05, ** p<0.01, ***p<0.001. HC (n=6), 1 (n=2), 2 (n=9) 3 (n=8).

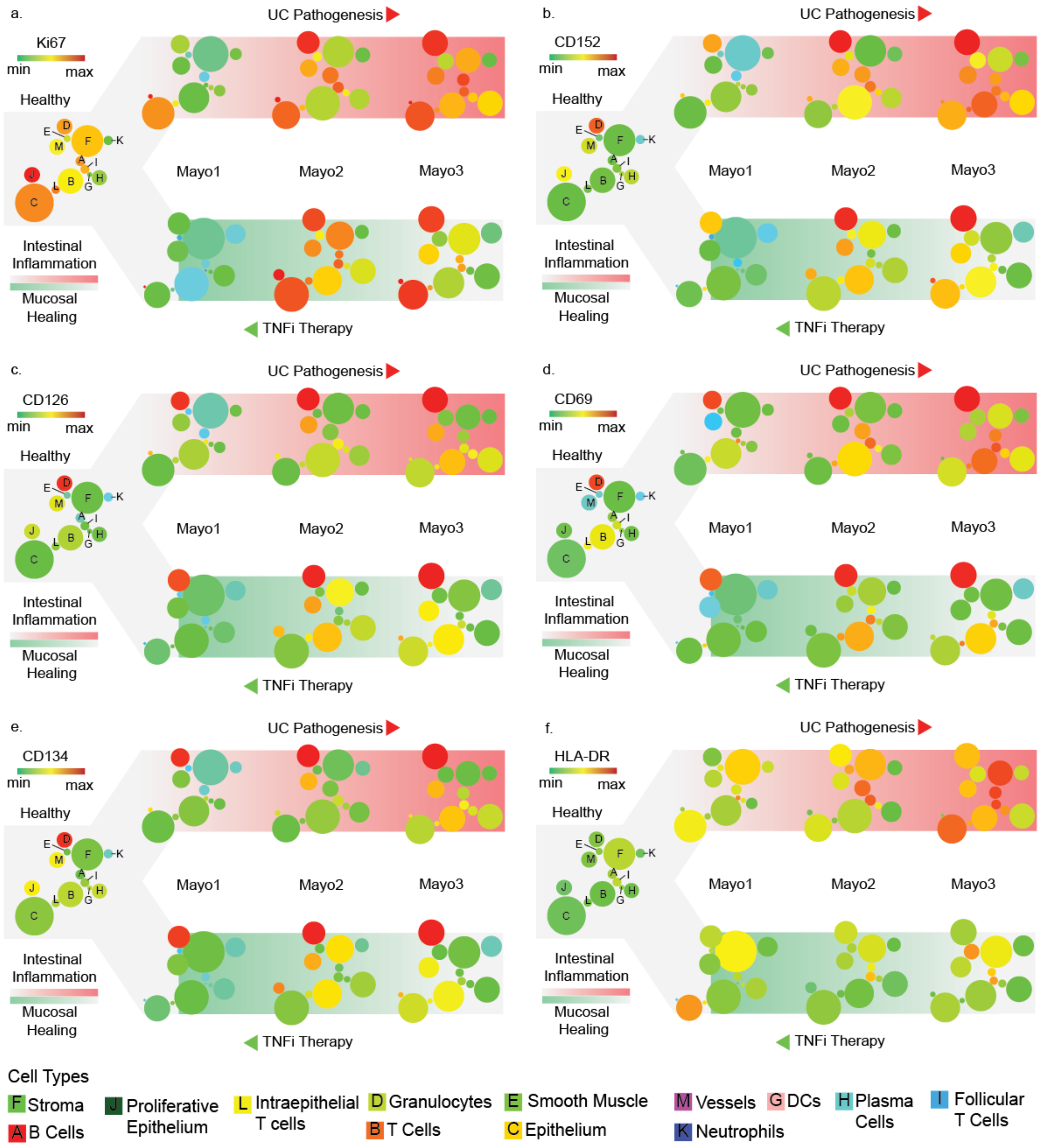


**Figure S6. Biomarker expression across cell populations and disease states. (a-f)** Scaffolds depicting minimum spanning tree representations of cell populations and expression of **a)** Ki67, **b)** CD152, **c)** CD126, **d)** CD69, **e)** CD134, and **f)** HLA-DR along trajectories of UC pathogenesis and healing during TNF therapy. Node size represents the average frequency of each cell population for each cohort and color represents the relative expression of indicated biomarker. Color scale: green-low, red-high.

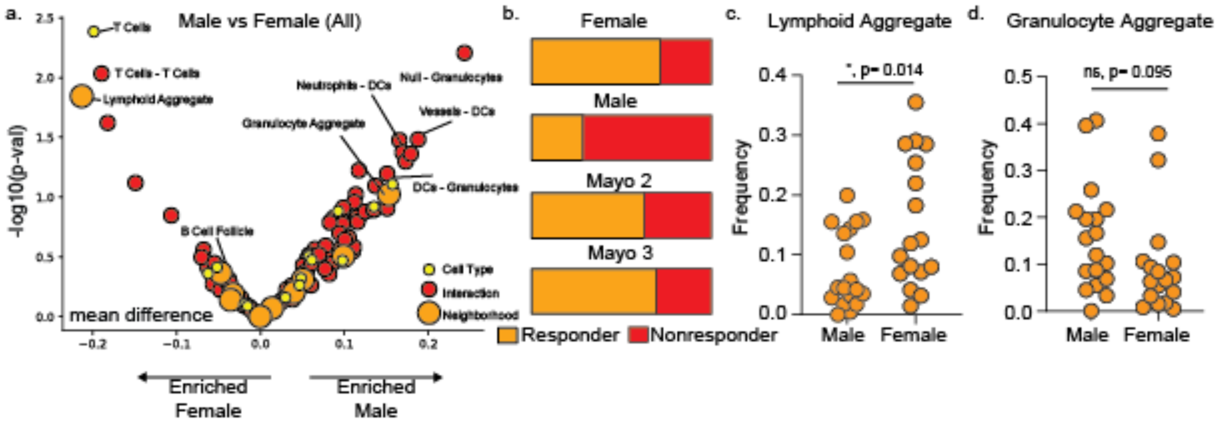


**Figure S7. Sex differences in UC. (a)** Volcano plot of cell types (yellow), cell contacts (red), and cellular neighborhoods (orange) between male and female UC patients. Points to the right are enriched in males, points to the left are enriched in females. **(b)** TNFi responders versus nonresponders, binned by sex and disease score. Female patients were more likely to respond than male patients. We did not observe substantial differences in responsiveness between patients with Mayo score of 2 versus Mayo score of 3. **(c-d)** Sex difference comparisons between top differential neighborhoods in patients with a Mayo score of 2. **c)** Female patients were enriched for lymphoid aggregates (p=0.014), which are part of the adaptive immune response. **d)** Male patients were enriched for granulocyte aggregates, part of the innate immune response (p=0.095).

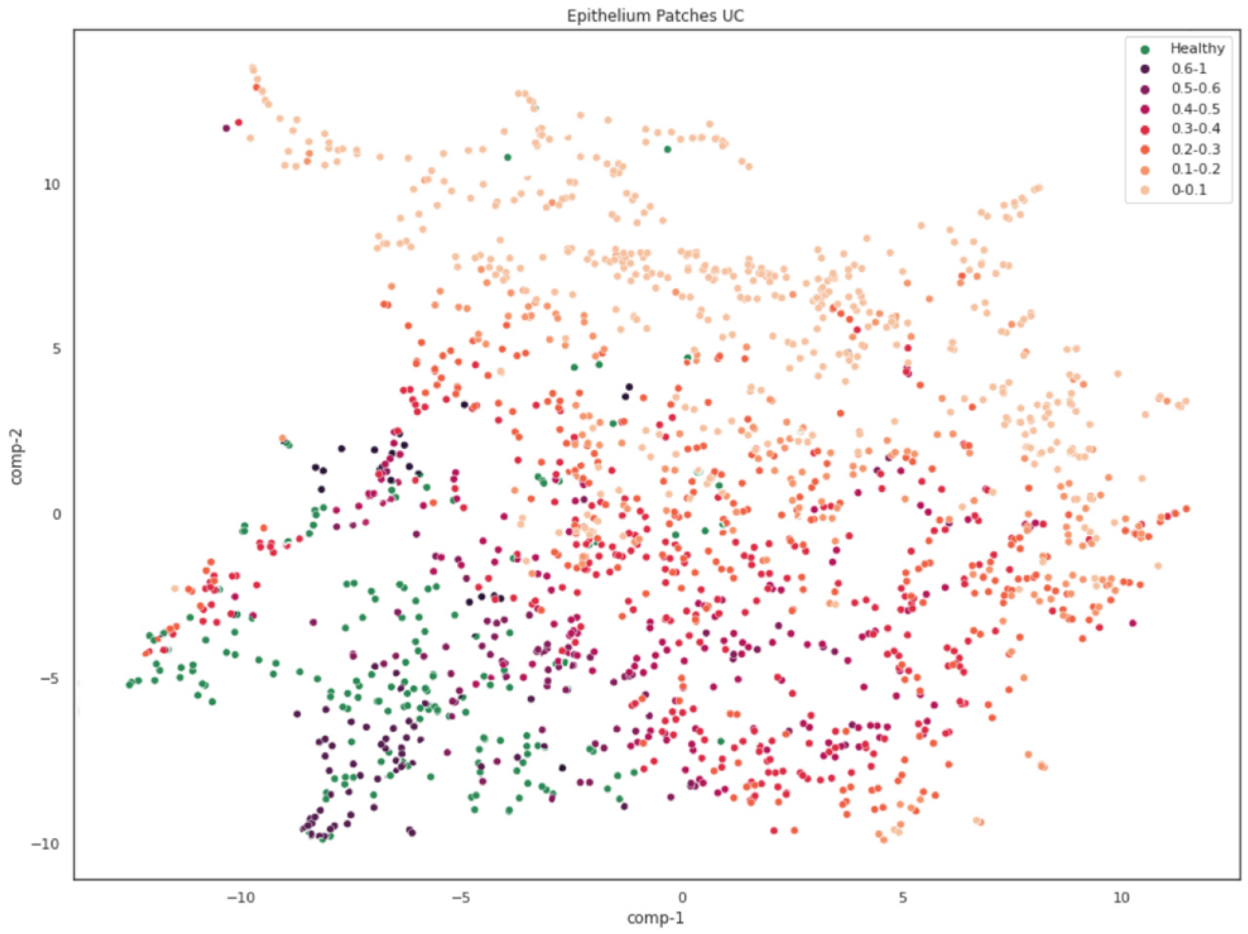


**Figure S8: tSNE plot of UC patients image patches compared with healthy patches.** UC patient patches (pink to purple color gradient) demonstrate substantial variability in comparison to image patches from healthy patients (green). As the epithelial ratio of the UC patches increase, they become increasingly similar to healthy patches though they still remain visibly distinct. This illustrates one reason why accounting for the variability in the patch-dependent representation and classification of UC patients, rather than using patient-averages, may result in better predictive performance.

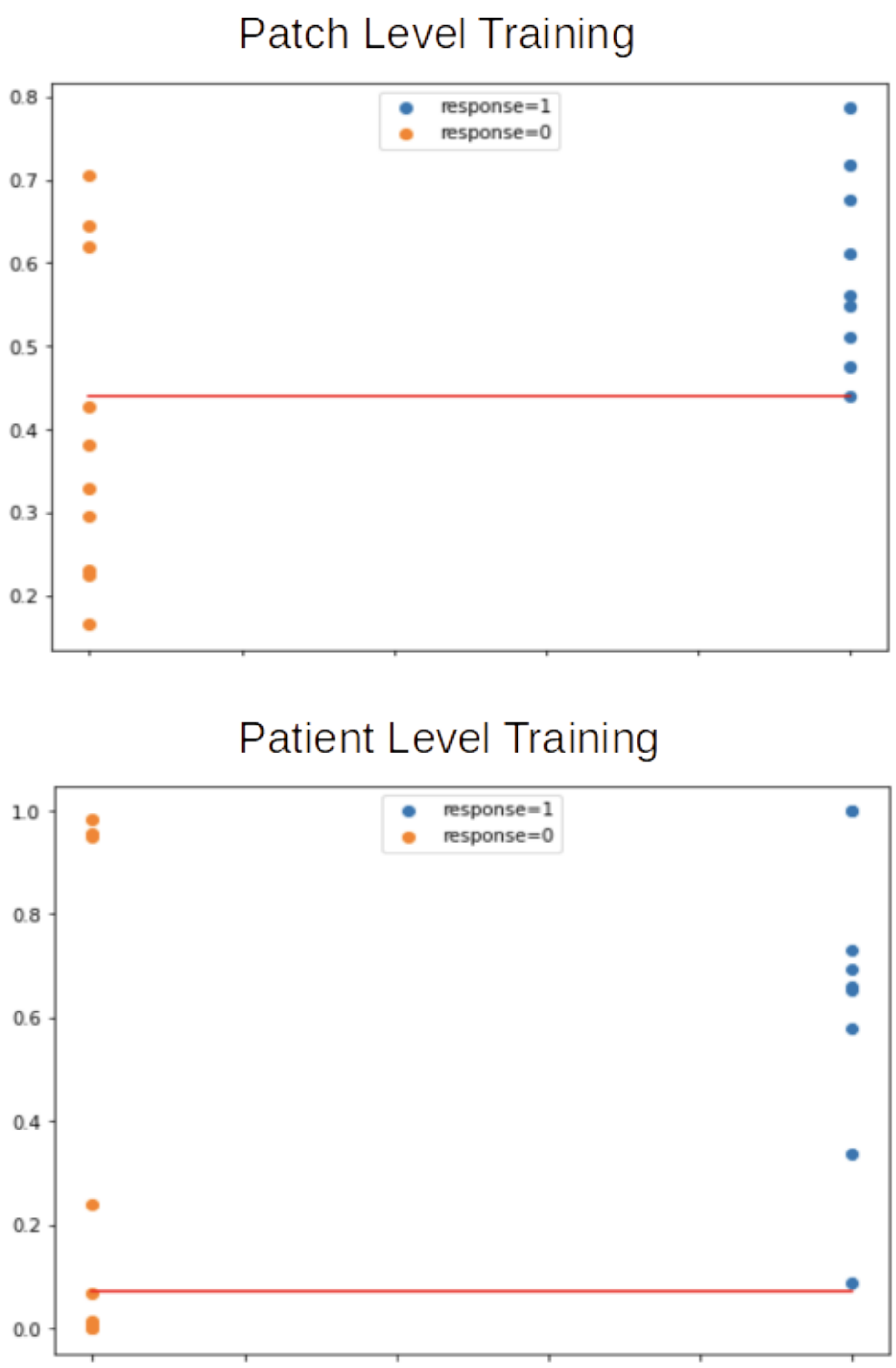


**Figure S9. Validation Patient-level Predictions for Patient vs Patch-level Training with CNN representation for severe (class 1) vs. mild/moderate (class 0).** Patient-level validation predictions for patch-level and patient-level training when using the CNN representation (ResNet-50 as pre-trained network and 1000x1000px patches as inputs). Patch-level training gives predictions that appear to behave more reasonably. The red line gives the threshold which maximizes Youden's Index on the validation set when weighting each patient inversely proportionally to the total number of patients belonging to the same class.

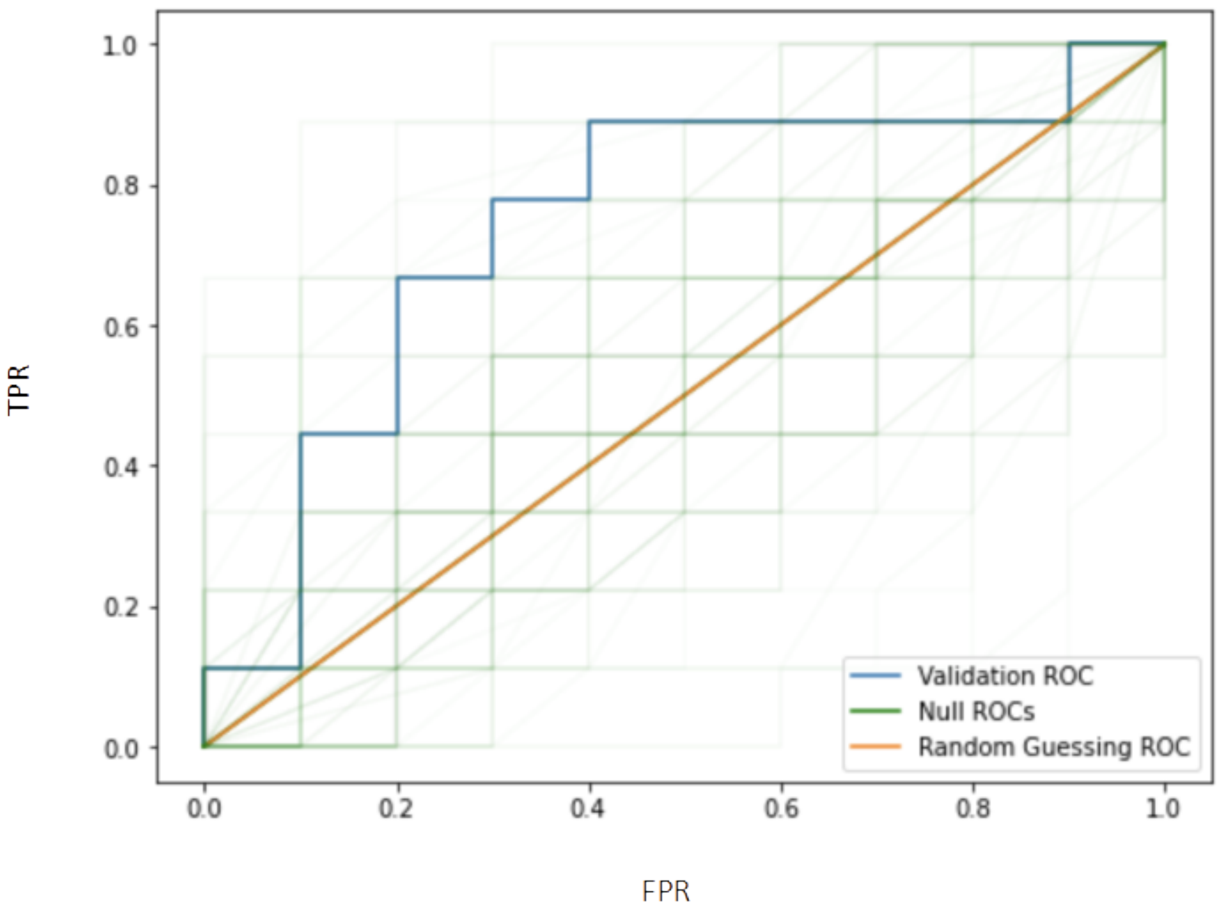


**Figure S10. Highest TNFi Patient-Level Validation AUROC: CNN representation (ResNet-50 pretrained network, 1000x1000px input patches) with Patient-Level Training.** The highest resulting validation AUROC over choices of pre-trained neural network, input patch size, and patient vs. patch-level training. The p-value from the permutation test is not significant (p=0.13).

## Supplemental Tables

**Table S1.** Patient demographics, medical characteristics, TNFi therapy outcomes, and tissue data collected through the Inflammatory Bowel Disease Biobank at the University of California, San Diego.

| Patient Characteristics | UC[a] | UC TNFi[b] | HC[c] |
|---|---|---|---|
| Patients (n) | 14 | 15 | 5 |
| *Female* | 7 | 8 | 1 |
| *Male* | 7 | 7 | 4 |
| *Age, years (mean ± SD)* | 39.9 ± 14.3 | 43.3 ± 18.2 | 65.2 ± 8.8 |
| *Age range, years* | 21 - 67 | 23 - 78 | 50 - 73 |
| Disease Characteristics | | | |
| *Disease duration, years (mean ± SD)* | 8.86 ± 10.8 | 7.2 ± 5.9 | --- |
| *Disease duration range, years* | 1 - 40 | 2 - 26 | --- |
| Colonic Segment Bx Location | | | |
| *Rectum* | 11 (78.6%) | 13 (86.7%) | 5 (100%) |
| *Right Colon* | 3 (21.4%) | 0 | 0 |
| *Left Colon* | 0 | 2 (13.3%) | 0 |
| Mayo ES Classification | | | |
| *Mayo Score 0* | 0 | 0 | --- |
| *Mayo Score 1* | 2 (14.3 %) | 2 (13.3%) | --- |
| *Mayo Score 2* | 7 (50%) | 6 (40%) | --- |
| *Mayo Score 3* | 5 (35.7%) | 7 (46.7%) | --- |
| TNFi Therapy Outcomes | | | |
| *Subsequent non-responders* | 3 (21.4%)* | 7 (46.7%) | --- |
| *Subsequent responders* | 3 (21.4%)* | 6 (40%) | --- |
| *N/A* | 8 (57.2%) | 2 (13.3%) | --- |

[a] Subjects labeled "UC" had not been treated with TNFi at time of biopsy. An asterisk indicates that the patient received TNFi therapy subsequently, and response was retrieved from the patient history. b Subjects labeled "UC TNFi" were being treated with TNFi at the time of biopsy. c Subjects labeled "HC" were controls without IBC.

**Table S2.** Antibody panel information

| Antigen | Clone(s) | Manufacturer | Catalog No. | CODEX oligo | Working dilution | Exposure time |
|---|---|---|---|---|---|---|
| Vimentin | RV202 | BD | 550513 | 7 | 1:100 | 1/3 s |
| CD85j | HP-F1 | ThermoFisher | 16-5129-82 | 8 | 1:100 | 1/3 s |
| CD15 | HI98 | Biolegend | 301902 | 15 | 1:100 | 1/6 s |
| CD21 | Bu32 | Biolegend | 354902 | 21 | 1:100 | 1/3 s |
| CD4 | A161A1 | Biolegend | 357402 | 28 | 1:100 | 1/3 s |
| CD66 | B1.1/CD66 | BD | 551354 | 41 | 1:100 | 1/4 s |
| CD8 | SK1 | Biolegend | 344702 | 43 | 1:100 | 1/3 s |
| CD11c | B-ly6 | BD | 555391 | 44 | 1:100 | 1/3 s |
| CD54 | HA58 | BD | 555510 | 46 | 1:100 | 1/1.2 s |
| CD7 | CD7-6B7 | Biolegend | 343102 | 58 | 1:100 | 1/3 s |
| TCRyd | B1 | Biolegend | 331202 | 63 | 1:100 | 1/3 s |
| CD38 | HB-7 | Biolegend | 356602 | 66 | 1:100 | 1/6 s |
| CD90 | 5 E10 | BD | 555593 | 68 | 1:100 | 1/5 s |
| CD40 | HB14 | Biolegend | 313002 | 70 | 1:100 | 1/1.5 s |
| CD45RA | HI100 | Biolegend | 304102 | 72 | 1:100 | 1/3 s |
| podoplanin | NC-08212.77 | Biolegend | 337002 | 32 | 1:100 | 1/3.5 s |
| collagen IV | ab6586 | Abcam | ab6586 | 33 | 1:100 | 1/15 s |

| | | | | | | |
|---|---|---|---|---|---|---|
| CD19 | HIB19 | Biolegend | 302202 | 2 | 1:100 | 1/3 s |
| TIGIT | A15153G | Biolegend | 372702 | 5 | 1:100 | 1/2 s |
| HLA-DR | TU36 | Biolegend | 361602 | 11 | 1:100 | 1/10 s |
| CD274 | 29E.2A3 | Biolegend | 329702 | 14 | 1:100 | 1/5 s |
| CD3 | UCHT1 | BD | 555330 | 20 | 1:100 | 1/3 s |
| CD69 | FN50 | Biolegend | 310902 | 24 | 1:100 | 1/6 s |
| CD57 | HCD57 | Biolegend | 359602 | 30 | 1:100 | 1/3 s |
| HIF-1a | 1A3 | Abcam | ab82832 | 36 | 1:100 | 1/1.2 s |
| CD34 | 561 | Biolegend | 343602 | 38 | 1:100 | 1/3 s |
| VCAM-1 | 51-10c9 | BD | 555645 | 48 | 1:100 | 1/2 s |
| CD36 | CB38 | ThermoFisher | MA1-10209 | 49 | 1:100 | 1/3 s |
| CD1c | L161 | Biolegend | 331502 | 55 | 1:100 | 1/3 s |
| CD45 | HI30 | Biolegend | 304002 | 56 | 1:100 | 1/20 s |
| FoxP3 | 236A/E7 | ThermoFisher | 14-4777-82 | 57 | 1:100 | 1/4 s |
| cytokeratin | C-11 | Biolegend | 628602 | 67 | 1:100 | 1/7.5 s |
| CD278 | C398.4A | Biolegend | 313502 | 71 | 1:100 | 1/3 s |
| CD117 | YB5.B8 | ThermoFisher | 14-1179-82 | 74 | 1:100 | 1/3 s |
| CD5 | UCHT2 | Biolegend | 300602 | 75 | 1:100 | 1/10 s |

| | | | | | | |
|---|---|---|---|---|---|---|
| CD31 | WM59 | Biolegend | 303102 | 3 | 1:100 | 1/4 s |
| Ki67 | B56 | BD | 556003 | 6 | 1:100 | 1/12 s |
| CD152 | BNI3 | BD | 555851 | 23 | 1:100 | 1/3 s |
| CD2 | RPA-2.10 | Biolegend | 300202 | 25 | 1:100 | 1/3 s |
| CD126 | BDm5 | BD | 551462 | 81 | 1:100 | 1/2 s |
| CD134 | BerACT35 | Biolegend | 350002 | 69 | 1:100 | 1/2 s |
| CD56 | B159 | BD | 555514 | 29 | 1:100 | 1/3 s |
| MMP12 | ab137444 | Abcam | ab137444 | 45 | 1:100 | 1/2.3 s |
| CD137 | b4-1 | Biolegend | 309802 | 53 | 1:100 | 1/2 s |
| CD49f | GoH3 | BD | 555734 | 51 | 1:100 | 1/3 s |
| CD16 | 3G8 | BD | 555404 | 52 | 1:100 | 1/3 s |
| CD123 | 656 | Biolegend | 306002 | 59 | 1:100 | 1/3 s |
| HLA-ABC | G46-2.6 | BD | 555551 | 60 | 1:100 | 1/3 s |
| CD127 | A019D5 | Biolegend | 351302 | 61 | 1:100 | 1/1.2 s |
| CD104 | 450-9D | BD | 555721 | 76 | 1:100 | 1/3 s |
| TNFR2 | BD-htnfr-m1 | BD | 551311 | 77 | 1:100 | 1/2 s |
| CD279 | EH12.2H7 | Biolegend | 329902 | 79 | 1:100 | 1/3 s |

**Table S3**. Reagents

| Reagent | Source | Identifier |
|---|---|---|
| **Antibodies and Proteins** | | |
| Purified antibodies, see **Table S2** | Various | Various |
| **Oligonucleotides** | | |
| CODEX oligonucleotides, see **Table S3** | TriLink Biotechnologies and Integrated DNA Technologies | N/A |
| **Biological Samples** | | |
| Fresh frozen tissue | University of California, San Diego | N/A |
| **Chemicals and Reagents** | | |
| PBS | Thermo Fisher Scientific | 14190-250 |
| NaCl | Thermo Fisher Scientific | S271-10 |
| $Na_2HPO_4$ | Sigma | S7907 |
| $NaH_2PO_4 \cdot 7\ H_2O$ | Sigma | S9390 |
| $MgCl_2 \cdot 6\ H_2O$ | Sigma | M2670 |
| $NaN_3$ | Sigma | S8032 |
| EDTA | Sigma | 93302 |
| TCEP | Sigma | C4706 |
| NaOH | Sigma | S8263 |
| BS3 | Thermo Fisher Scientific | 21580 |

| | | |
|---|---|---|
| Poly-L-Lysine | Sigma | P8920 |
| DMSO | Thermo Fisher Scientific | D128-4 |
| DMSO | Sigma | 472301 |
| DMSO ampoules | Sigma | D2650 |
| Paraformaldehyde ampoules, 16% | Thermo Fisher Scientific | 50-980-487 |
| BSA | Sigma | A3059 |
| Tris 1 M, pH 8.0 | Teknova | T1080 |
| Candor PBS antibody stabilizer solution | Thermo Fisher Scientific | NC0436689 |
| Salmon sperm DNA, sheared | Thermo Fisher Scientific | AM9680 |
| TritonTM X-100 | Sigma | T8787 |
| Ethanol, 100% | Sigma | E7023 |
| Acetone, 100% | Thermo Fisher Scientific | A929-4 |
| Methanol, 100% | Thermo Fisher Scientific | A412-4 |
| Trizma® HCl | Sigma | T3253 |
| Trizma® Base | Sigma | T1503 |
| Drierite indicating desiccant | Thermo Fisher Scientific | 07-578-3A |

| | | |
|---|---|---|
| Bondic polyacrylamide gel | Amazon | B018IBEHQU |
| Antibody diluent | Agilent | S080981-2 |
| Protein block, serum-free | Agilent | X090930-2 |
| Dual endogenous enzyme-blocking reagent | Agilent | S200380-2 |
| Hematoxylin, ready-to-use | Agilent | S330930-2 |
| Eosin Y solution | Sigma | HT110116 |
| Cytoseal XYL | Thermo Fisher Scientific | 8312-4 |
| Sally Hansen Nail Polish, clear | Amazon | B00CMFMYEG |

**Critical Commercial Instruments, Consumables, Kits and Assays**

| | | |
|---|---|---|
| LTS filter tips, 10 µl | Rainin | 30389225 |
| LTS filter tips, 200 µl | Rainin | 30389239 |
| LTS filter tips, 1000 µl | Rainin | 30389212 |
| Amicon™ Ultra Centrifugal Filters, 50kDa | Thermo Fisher Scientific | UFC505096 |
| Nalgene™ Rapid Flow 500 ml filter, 0.2 µm | Thermo Fisher Scientific | 09-740-28C |
| Glass coverslips, 22x22 mm, # 1 1/2 | Electron Microscopy Sciences | 72204-01 |

| | | |
|---|---|---|
| Frosted microscope slides | Thermo Fisher Scientific | 12-550-343 |
| Glass coverslip storage box | Qintay | CS-22 |
| 22x22 mm coverslip mounting gaskets | Qintay | TMG-22 |
| Wheaton$^{TM}$ Coverslip glass jars | Thermo Fisher Scientific | 02-912-637 |
| Dumont #5/45 coverslip forceps | Fine Science Tools | 11251-33 |
| 8-strip tubes, 0.2 ml | E&K Scientific | 280008 |
| 8-strip caps, flat top | E&K Scientific | 491008 |
| 8-strip caps, dome top | E&K Scientific | 491018 |
| CODEX acrylic plates | Bayview Plastic Solutions | custom made |
| BZ-X710 fluorescence microscope | Keyence | N/A |
| Hoechst 33342 | Thermo Fisher Scientific | 62249 |
| DRAQ5 | Cell Signaling Technology | 4084L |
| CorningTM clear 96-well plates | Thermo Fisher Scientific | 07-200-762 |
| Axygen aluminum sealing film | VWR Scientific | 47734-817 |
| CODEX System | Akoya Biosciences | N/A |

**Software and Algorithms**

| | | |
|---|---|---|
| BZ-X viewer | Keyence | N/A |
| CODEX driver | Akoya Biosciences | N/A |
| CODEX Toolkit, version 1.3.5 | https://github.com/nolanlab/CODEX | (*27*) |
| Microvolution software for deconvolution | www.microvolution.com | N/A |
| ImageJ (Fiji version 2.0.0) | http://imagej.net | N/A |
| VorteX (X-shift clustering algorithm) | https://github.com/nolanlab/VORTEX | (*43*) |
| CellEngine | www.cellengine.com | (*44*) |
| R, version 3.4.3 | www.r-project.org | N/A |
| R studio desktop, version 1.1.423 | www.rstudio.com | N/A |
| Neighborhood analysis notebooks | https://github.com/nolanlab | (*28*) |
| Tensorly Python package | http://tensorly.org/ | (*45*) |
| Statsmodel Python package | https://www.statsmodels.org/ | (*46*) |
| Scikit learn Python package | https://scikit-learn.org/ | (*47*) |

| | | |
|---|---|---|
| Survival R package | https://cran.r-project.org/web/packages/ survival/index.html | (*48*) |
| Glmnet R package | https://cran.r-project.org/web/packages/ glmnet/index.html | (*49*) |
| Visreg R package | https://cran.r-project.org/web/packages/ visreg/index.html | (*50*) |
| Deldir R package | https://cran.r-project.org/web/packages/ deldir/index.html | N/A |
| ComplexHeatmap R package | release/bioc/html/ ComplexHeatmap.html | (*51*) |
| The Human Protein Atlas | www.proteinatlas.org | N/A |
| Pathology Outlines | www.pathologyoutlines.com | N/A |

| **<u>Stock Solutions</u>** | **Composition** |
|---|---|
| Staining solution 1 (S1) | 5 mM EDTA, 0.5% w/v bovine serum albumin, and 0.02% w/v $NaN_3$ in PBS, store at 4 °C. |
| Staining solution 2 (S2) | 61 mM $NaH_2PO_4 \cdot 7\ H_2O$, 39 mM $NaH_2PO_4$, and 250 mM NaCl in a 1:0.7 v/v mix of **S1** and double-distilled (dd)$H_2O$; final pH 6.8-7.0. |
| Staining solution 4 (S4) | 0.5 M NaCl in **S1**. |
| TE Buffer | 10 mM Tris pH 8.0, 1 mM EDTA and 0.02% w/v $NaN_3$ in dd$H_2O$. |
| Tris buffer | 50 mM, pH 7.2 (at room temperature/25 °C) was prepared using 7.02 g/L Trizma HCl and 0.67 g/L Trizma base in dd$H_2O$. |
| Conjugation buffer C (C) | 150 mM NaCl, 2 mM **Tris buffer solution**, pH 7.2, 1 mM EDTA and 0.02% w/v $NaN_3$ in ddH2O |

| | |
|---|---|
| Codex 2.0 buffer (H2) | 150 mM NaCl, 10 mM Tris, pH 7.5, 10 mM $MgCl_2 \cdot 6\ H_2O$, 0.1% w/vTriton X-100, and 0.02% w/v $NaN_3$ in $ddH_2O$. [DRH1] |
| Blocking reagent 1 (B1) | 1 mg/ml mouse IgG (Sigma) in **S2**. |
| Blocking reagent 2 (B2) | 1 mg/ml rat IgG (Sigma) in **S2**. |
| Blocking reagent 3 (B3) | Sheared salmon sperm DNA, 10 mg/ml (Thermo Fisher). |
| Blocking reagent 4 (B4) | Mixture of 57 non-modified CODEX oligonucleotides (see **Table S2**) at a final concentration of 0.5 mM each. |
| BS3 fixative solution (BS3) | 200 mg/ml BS3 in DMSO, fresh, stored at -20 °C in 15 µl aliquots. |
| TCEP solution | 0.5 M TCEP in $ddH_2O$, pH 7.0. |
| Rendering buffer | 20% DMSO (v/v) in **H2 buffer**. |
| Stripping buffer | 80% DMSO (v/v) in **H2 buffer**. |

**Table S4.** Patient-level validation AUROC and patient-level validation accuracy for all three prediction tasks for both standard and CNN representations, where for CNN representations we vary the pre-trained neural network and input patch size. UC corresponds to UC (class 1) versus healthy (class 0), MAYO corresponds to severe (class 1) vs. mild/moderate (class 0), and TNFi corresponds to responder (class 1) vs. resistor (class 0). We identify a particular CNN representation by its pre-trained neural network architecture and input patch size. When reporting misclassifications we give the number of false positives (fp) and false negatives (fn). Misclassifications are not given for the CNN representation with input patch size 500x500px due to computational constraints. Optimal number of misclassifications comes from classifying according to the threshold which maximizes Youden's Index on the validation set when weighting each patient inversely proportionally to the total number of patients belonging to the same class.

| | UC Patient-level Training | | | UC Patch-level Training | | |
|---|---|---|---|---|---|---|
| Representation | AUC | # of Misclass. (fp/fn) | Optimal # of Misclass. (fp/fn) | AUC | # of Misclass. (fp/fn) | Optimal # of Misclass. (fp/fn) |
| Standard | 0.98 | 0/1 | 0/1 | 0.98 | 2/1 | 0/1 |
| ResNet-18 500x500px | 0.95 | | 0/1 | 0.96 | | 0/1 |
| ResNet-18 1000x1000px | 0.95 | 0/1 | 0/1 | 1.00 | 0/1 | 0/0 |
| ResNet-50 500x500px | 0.97 | | 0/2 | 0.96 | | 0/1 |
| ResNet-50 1000x1000px | 0.96 | 0/3 | 0/2 | 0.95 | 0/2 | 0/1 |
| ResNet-152 500x500px | 0.98 | | 0/2 | 0.96 | | 0/1 |
| ResNet-152 1000x1000px | 0.98 | 0/1 | 0/1 | 0.98 | 0/1 | 0/1 |
| Shufflenet 500x500px | 0.96 | | 0/2 | 0.95 | | 0/1 |
| Shufflenet 1000x1000px | 0.95 | 0/3 | 0/2 | 0.95 | 0/2 | 0/1 |
| | | | | | | |
| | MAYO Patient-level Training | | | MAYO Patch-level Training | | |
| Representation | AUC | # of Misclass. (fp/fn) | Optimal # of Misclass. (fp/fn) | AUC | # of Misclass. (fp/fn) | Optimal # of Misclass. (fp/fn) |
| Standard | 0.80 | 4/1 | 3/0 | 0.84 | 3/4 | 4/0 |
| ResNet-18 500x500px | 0.74 | | 3/1 | 0.78 | | 4/0 |
| ResNet-18 1000x1000px | 0.76 | 5/1 | 5/0 | 0.81 | 4/1 | 4/0 |
| ResNet-50 | 0.73 | | 5/0 | 0.77 | | 3/1 |

| | | | | | | |
|---|---|---|---|---|---|---|
| 500x500px | | | | | | |
| ResNet-50 1000x1000px | 0.76 | 4/0 | 4/0 | 0.79 | 4/1 | 3/0 |
| ResNet-152 500x500px | 0.74 | | 4/1 | 0.81 | | 4/0 |
| ResNet-152 1000x1000px | 0.72 | 4/3 | 4/1 | 0.82 | 4/1 | 4/0 |
| Shufflenet 500x500px | 0.63 | | 4/1 | 0.71 | | 4/1 |
| Shufflenet 1000x1000px | 0.63 | 5/1 | 5/1 | 0.77 | 5/1 | 4/0 |
| | | | | | | |
| | TNF Patient-level Training | | | TNF Patch-level Training | | |
| Representation | AUC | # of Misclass. (fp/fn) | Optimal # of Misclass. (fp/fn) | AUC | # of Misclass. (fp/fn) | Optimal # of Misclass. (fp/fn) |
| Standard | 0.46 | 2/9 | 2/5 | 0.49 | 1/6 | 2/5 |
| ResNet-18 500x500px | 0.47 | | 2/6 | 0.48 | | 0/7 |
| ResNet-18 1000x1000px | 0.53 | 6/3 | 5/2 | 0.51 | 2/7 | 0/7 |
| ResNet-50 500x500px | 0.74 | | 1/4 | 0.64 | | 3/3 |
| ResNet-50 1000x1000px | 0.74 | 2/2 | 4/1 | 0.66 | 1/5 | 1/4 |
| ResNet-152 500x500px | 0.61 | | 3/3 | 0.59 | | 3/4 |
| ResNet-152 1000x1000px | 0.61 | 5/2 | 6/1 | 0.64 | 4/5 | 5/2 |
| Shufflenet 500x500px | 0.46 | | 4/4 | 0.56 | | 3/4 |
| Shufflenet 1000x1000px | 0.49 | 3/7 | 1/6 | 0.54 | 2/6 | 3/4 |

## REFERENCES


1. R. Ungaro, S. Mehandru, P. B. Allen, L. Peyrin-Biroulet, J.-F. Colombel, Ulcerative colitis. *Lancet* **389**, 1756–1770 (2017).

2. G. S. Cooper, M. L. K. Bynum, E. C. Somers, Recent insights in the epidemiology of autoimmune diseases: Improved prevalence estimates and understanding of clustering of diseases. *J. Autoimmun.* **33**, 197–207 (2009).

3. M. F. Neurath, Targeting immune cell circuits and trafficking in inflammatory bowel disease. *Nat. Immunol.* **20**, 970–979 (2019).

4. E. Dekker, P. J. Tanis, J. L. A. Vleugels, P. M. Kasi, M. B. Wallace, Colorectal cancer. *Lancet* **394**, 1467–1480 (2019).

5. O. Olén, R. Erichsen, M. C. Sachs, L. Pedersen, J. Halfvarson, J. Askling, A. Ekbom, H. T. Sørensen, J. F. Ludvigsson, Colorectal cancer in ulcerative colitis: A Scandinavian population-based cohort study. *Lancet Gastroenterol. Hepatol.* **395**, 123–131 (2020).

6. L. A. Sceats, A. M. Morris, M. K. Bundorf, K. Park, C. Kin, Sex differences in treatment strategies among patients with ulcerative colitis: A retrospective cohort analysis of privately insured patients. *Dis. Colon Rectum* **62**, 586–594 (2019).

7. Y. Wang, Q. Ouyang; APDW 2004 Chinese IBD working group, Ulcerative colitis in China: Retrospective analysis of 3100 hospitalized patients. *J. Gastroenterol. Hepatol.* **22**, 1450–1455 (2007).

8. J. Bábíčková, Ľ. Tóthová, E. Lengyelová, A. Bartoňová, J. Hodosy, R. Gardlík, P. Celec, Sex differences in experimentally induced colitis in mice: A role for estrogens. *Inflammation* **38**, 1996–2006 (2015).

9. M. F. Neurath, Current and emerging therapeutic targets for IBD. *Nat. Rev. Gastroenterol. Hepatol.* **14**, 269–278 (2017).

10. J. D. Feuerstein, K. L. Isaacs, Y. Schneider, S. M. Siddique, Y. Falck-Ytter, S. Singh, K. Chachu, L. Day, B. Lebwohl, T. Muniraj, A. Patel, A. F. Peery, R. Shah, S. Sultan, H. Singh, S. Singh, S. Spechler, G. Su, A. P. Thrift, J. M. Weiss, A. V. Weizman, J. Feuerstein, S. Singh, K. Isaacs, Y. Schneider, Y. Falck-Ytter, S. M. Siddique, J. Allegretti, J. Terdiman, S. Singh, S. M. Siddique, AGA clinical practice guidelines on the management of moderate to severe ulcerative colitis. *Gastroenterology* **158**, 1450–1461 (2020).

11. J. E. Axelrad, S. Lichtiger, V. Yajnik, Inflammatory bowel disease and cancer: The role of inflammation, immunosuppression, and cancer treatment. *World J. Gastroenterol.* **22**, 4794–4801 (2016).

12. U. Kopylov, E. Seidman, Predicting durable response or resistance to antitumor necrosis factor therapy in inflammatory bowel disease. *Therap. Adv. Gastroenterol.* **9**, 513–526 (2016).

13. S. Ben-Horin, Y. Chowers, Tailoring anti-TNF therapy in IBD: Drug levels and disease activity. *Nat. Rev. Gastroenterol. Hepatol.* **11**, 243–255 (2014).

14. C. Abraham, P. S. Dulai, S. Vermeire, W. J. Sandborn, Lessons learned from trials targeting cytokine pathways in patients with inflammatory bowel diseases. *Gastroenterology* **152**, 374–388.e4 (2017).

15. R. Gaujoux, E. Starosvetsky, N. Maimon, F. Vallania, H. Bar-Yoseph, S. Pressman, R. Weisshof, I. Goren, K. Rabinowitz, M. Waterman, H. Yanai, I. Dotan, E. Sabo, Y. Chowers, P. Khatri, S. S. Shen-Orr; Israeli IBD research Network (IIRN), Cell-centred meta-analysis reveals baseline predictors of anti-TNFα non-response in biopsy and blood of patients with IBD. *Gut* **68**, 604–614 (2019).

16. J.-F. Colombel, B. E. Sands, P. Rutgeerts, W. Sandborn, S. Danese, G. D'Haens, R. Panaccione, E. V. Loftus, S. Sankoh, I. Fox, A. Parikh, C. Milch, B. Abhyankar, B. G. Feagan, The safety of vedolizumab for ulcerative colitis and Crohn's disease. *Gut* **66**, 839–851 (2017).

17. S. P. Borg-Bartolo, R. K. Boyapati, J. Satsangi, R. Kalla, Precision medicine in inflammatory bowel disease: Concept, progress and challenges. *F1000Res* **9**, 54 (2020).

18. F. S. Lehmann, E. Burri, C. Beglinger, The role and utility of faecal markers in inflammatory bowel disease. *Therap. Adv. Gastroenterol.* **8**, 23–36 (2015).

19. N. S. Ding, A. Hart, P. D. Cruz, Systematic review: Predicting and optimising response to anti-TNF therapy in Crohn's disease–Algorithm for practical management. *Aliment. Pharmacol. Ther.* **43**, 30–51 (2016).

20. S. J. S. Rubin, L. Bai, Y. Haileselassie, G. Garay, C. Yun, L. Becker, S. E. Streett, S. R. Sinha, A. Habtezion, Mass cytometry reveals systemic and local immune signatures that distinguish inflammatory bowel diseases. *Nat. Commun.* **10**, 2686 (2019).

21. J. C. Martin, C. Chang, G. Boschetti, R. Ungaro, M. Giri, J. A. Grout, K. Gettler, L. Chuang, S. Nayar, A. J. Greenstein, M. Dubinsky, L. Walker, A. Leader, J. S. Fine, C. E. Whitehurst, M. L. Mbow, S. Kugathasan, L. A. Denson, J. S. Hyams, J. R. Friedman, P. T. Desai, H. M. Ko, I. Laface, G. Akturk, E. E. Schadt, H. Salmon, S. Gnjatic, A. H. Rahman, M. Merad, J. H. Cho, E. Kenigsberg, Single-cell analysis of Crohn's disease lesions identifies a pathogenic cellular module associated with resistance to anti-TNF therapy. *Cell* **178**, 1493–1508.e20 (2019).

22. Y. Haberman, R. Karns, P. J. Dexheimer, M. Schirmer, J. Somekh, I. Jurickova, T. Braun, E. Novak, L. Bauman, M. H. Collins, A. Mo, M. J. Rosen, E. Bonkowski, N. Gotman, A. Marquis, M. Nistel, P. A. Rufo, S. S. Baker, C. G. Sauer, J. Markowitz, M. D. Pfefferkorn, J. R. Rosh, B. M. Boyle, D. R. Mack, R. N. Baldassano, S. Shah, N. S. Leleiko, M. B. Heyman, A. M. Grifiths, A. S. Patel, J. D. Noe, B. J. Aronow, S. Kugathasan, T. D. Walters, G. Gibson, S. D. Thomas, K. Mollen, S. Shen-Orr, C. Huttenhower, R. J. Xavier, J. S. Hyams, L. A. Denson, Ulcerative colitis mucosal transcriptomes reveal mitochondriopathy and personalized mechanisms underlying disease severity and treatment response. *Nat. Commun.* **10**, 38 (2019).

23. H. W. Jackson, J. R. Fischer, V. R. T. Zanotelli, H. R. Ali, R. Mechera, S. D. Soysal, H. Moch, S. Muenst, Z. Varga, W. P. Weber, B. Bodenmiller, The single-cell pathology landscape of breast cancer. *Nature* **578**, 615–620 (2020).

24. N. Damond, S. Engler, V. R. T. Zanotelli, D. Schapiro, C. H. Wasserfall, I. Kusmartseva, H. S. Nick, F. Thorel, P. L. Herrera, M. A. Atkinson, B. Bodenmiller, A map of human type 1 diabetes progression by imaging mass cytometry. *Cell Metab.* **29**, 755–768.e5 (2019).

25. B. Bodenmiller, Multiplexed epitope-based tissue imaging for discovery and healthcare applications. *Cell Syst.* **2**, 225–238 (2016).

26. A. Kondo, S. Ma, M. Y. Y. Lee, V. Ortiz, D. Traum, J. Schug, B. Wilkins, N. A. Terry, H. Lee, K. H. Kaestner, Highly multiplexed image analysis of intestinal tissue sections in patients with inflammatory bowel disease. *Gastroenterology* **161**, 1940–1952 (2021).

27. Y. Goltsev, N. Samusik, J. Kennedy-Darling, S. Bhate, M. Hale, G. Vazquez, S. Black, G. P. Nolan, Deep profiling of mouse splenic architecture with CODEX multiplexed imaging. *Cell* **174**, 968–981.e15 (2018).

28. C. M. Schürch, S. S. Bhate, G. L. Barlow, D. J. Phillips, L. Noti, I. Zlobec, P. Chu, S. Black, J. Demeter, D. R. McIlwain, S. Kinoshita, N. Samusik, Y. Goltsev, G. P. Nolan, Coordinated cellular neighborhoods orchestrate antitumoral immunity at the colorectal cancer invasive front. *Cell* **183**, 838 (2020).

29. D. Phillips, M. Matusiak, B. R. Gutierrez, S. S. Bhate, G. L. Barlow, S. Jiang, J. Demeter, K. S. Smythe, R. H. Pierce, S. P. Fling, N. Ramchurren, M. A. Cheever, Y. Goltsev, R. B. West, M. S. Khodadoust, Y. H. Kim, C. M. Schürch, G. P. Nolan, Immune cell topography predicts response to PD-1 blockade in cutaneous T cell lymphoma. *Nat. Commun.* **12**, 6726 (2021).

30. S. Fine, K. Papamichael, A. S. Cheifetz, Etiology and management of lack or loss of response to anti-tumor necrosis factor therapy in patients with inflammatory bowel disease. **15**, 656–665 (2019).

31. H. Rabe, M. Malmquist, C. Barkman, S. Östman, I. Gjertsson, R. Saalman, A. E. Wold, Distinct patterns of naive, activated and memory T and B cells in blood of patients with ulcerative colitis or Crohn's disease. *Clin. Exp. Immunol.* **197**, 111–129 (2019).

32. K. L. Hyrich, K. D. Watson, A. J. Silman, D. P. M. Symmons; British Society for Rheumatology Biologics Register, Predictors of response to anti-TNF- therapy among patients with rheumatoid arthritis: Results from the British Society for Rheumatology Biologics Register. *Rheumatology* **45**, 1558–1565 (2006).

33. A. Davidson, B. Diamond, Autoimmune diseases. *N. Engl. J. Med.* **345**, 340–350 (2001).

34. T. M. Hunter, N. N. Boytsov, X. Zhang, K. Schroeder, K. Michaud, A. B. Araujo, Prevalence of rheumatoid arthritis in the United States adult population in healthcare claims databases, 2004–2014. *Rheumatol. Int.* **37**, 1551–1557 (2017).

35. B. S. Boland, Z. He, M. S. Tsai, J. G. Olvera, K. D. Omilusik, H. G. Duong, E. S. Kim, A. E. Limary, W. Jin, J. J. Milner, B. Yu, S. A. Patel, T. L. Louis, T. Tysl, N. S. Kurd, A. Bortnick, L. K. Quezada, J. N. Kanbar, A. Miralles, D. Huylebroeck, M. A. Valasek, P. S. Dulai, S. Singh, L.-F. Lu, J. D. Bui, C. Murre, W. J. Sandborn, A. W. Goldrath, G. W. Yeo, J. T. Chang, Heterogeneity and clonal relationships of adaptive immune cells in ulcerative colitis revealed by single-cell analyses. *Sci. Immunol.* **5**, eabb4432 (2020).

36. S. D. Rustgi, M. Kayal, S. C. Shah, Sex-based differences in inflammatory bowel diseases: A review. *Therap. Adv. Gastroenterol.* **13**, 1756284820915043 (2020).

37. G. S. Cooper, B. C. Stroehla, The epidemiology of autoimmune diseases. *Autoimmun. Rev.* **2**, 119–125 (2003).

38. O. Wéra, P. Lancellotti, C. Oury, The dual role of neutrophils in inflammatory bowel diseases. *J. Clin. Med.* **5**, 118 (2016).

39. M. G. Buell, M. C. Berin, Neutrophil-independence of the initiation of colonic injury. Comparison of results from three models of experimental colitis in the rat. *Dig. Dis. Sci.* **39**, 2575–2588 (1994).

40. M. Natsui, K. Kawasaki, H. Takizawa, S. I. Hayashi, Y. Matsuda, K. Sugimura, K. Seki, R. Narisawa, F. Sendo, H. Asakura, Selective depletion of neutrophils by a monoclonal antibody, RP-3, suppresses dextran sulphate sodium-induced colitis in rats. *J. Gastroenterol. Hepatol.* **12**, 801–808 (1997).

41. J. Satsangi, M. S. Silverberg, S. Vermeire, J.-F. Colombel, The Montreal classification of inflammatory bowel disease: Controversies, consensus, and implications. *Gut* **55**, 749–753 (2006).

42. M. Saerens, P. Latinne, C. Decaestecker. Adjusting the outputs of a classifier to new a priori probalities: A simple procedure. *Neural Comput.* **14**, 21–41 (2002).

43. N. Samusik, Z. Good, M. H. Spitzer, K. L. Davis, G. P. Nolan, Automated mapping of phenotype space with single-cell data. *Nat. Methods* **13**, 493–496 (2016).

44. Z. B. Bjornson-Hooper, G. K. Fragiadakis, M. H. Spitzer, D. Madhireddy, K. Hu, K. Lundsten, G. P. Nolan, Cell type-specific monoclonal antibody cross-reactivity screening in non-human primates and development of comparative immunophenotyping panels for CyTOF. *bioRxiv*, 577759 (2019).

45. J. Kossaifi, Y. Panagakis, A. Anandkumar, M. Pantic, TensorLy: Tensor learning in Python. 10.48550/arXiv.1610.09555 (2018).

46. S. Seabold, J. Perktold, Statsmodels: Econometric and statistical modeling with python, in *Proceedings of the 9th Python in Science Conference* (SciPy, 2010), Austin, Texas, 28 June to 3 July 2010, pp. 92–96.

47. F. Pedregosa, G. Varoquaux, A. Gramfort, V. Michel, B. Thirion, O. Grisel, M. Blondel, P. Prettenhofer, R. Weiss, V. Dubourg, J. Vanderplas, A. Passos, D. Cournapeau, M. Brucher, M. Perrot, É. Duchesnay, Scikit-learn: Machine learning in Python. *J. Mach. Learn. Res.* **12**, 2825–2830 (2011).

48. T. M. Therneau, T. L. (original S.->R port and R. maintainer until 2009), A. Elizabeth, C. Cynthia, Survival: Survival analysis (2022); https://CRAN.R-project.org/package=survival.

49. J. Friedman, T. Hastie, R. Tibshirani, Regularization paths for generalized linear models via coordinate descent. *J. Stat. Softw.* **33**, 1–22 (2010).

50. P. Breheny, W. Burchett, Visualization of regression models using visreg. *R J.* **9**, 56–71 (2017).

51. Z. Gu, R. Eils, M. Schlesner, Complex heatmaps reveal patterns and correlations in multidimensional genomic data. *Bioinformatics* **32**, 2847–2849 (2016).
